# Determination of Metallic Impurities by ICP-MS Technique in Eyeshadows Purchased in Poland. Part I

**DOI:** 10.3390/molecules26216753

**Published:** 2021-11-08

**Authors:** Aleksandra Pawlaczyk, Magdalena Gajek, Martyna Balcerek, Małgorzata I. Szynkowska-Jóźwik

**Affiliations:** Faculty of Chemistry, Institute of General and Ecological Chemistry, Lodz University of Technology, Zeromskiego 116, 90-924 Lodz, Poland; magdalena.gajek@edu.p.lodz.pl (M.G.); martyna.balcerek1@wp.pl (M.B.); malgorzata.szynkowska@p.lodz.pl (M.I.S.-J.)

**Keywords:** eye shadows, make-up cosmetics, metals, ICP-MS, PCA, CA, color, brand, country, price, regulations

## Abstract

Eye shadows, which are products willingly and frequently used by women and even children, have been reported in literature to contain toxic metals. In this work, a total of 94 eye shadows samples available on the Polish market were collected. Eye shadow products have been selected in order to include several parameters important from the point of view of the typical consumer such as: product type (mat/pearl), consumer group (for adults and children), price range (very cheap, medium price, expensive and very expensive), color (twelve different colors were tested), manufacturer (eight brands were investigated) or country of production (four countries were included). The concentration of selected metals (Ag, Ba, Bi, Cd, Pb, Sr, Tl) was determined by ICP-MS technique after the sample extraction with a mixture of nitric acid and hydrogen peroxide in a microwave closed system. For Ag, Cd and Tl, some results were below the established limit of quantification for the employed technique. The presence of strontium, barium, lead and bismuth was confirmed in all studied samples. The obtained results for analyzed elements were, in general, quite comparable with the data reported by other authors. A small number of samples exceeding the permissible values (two samples were beyond the limit value for Cd of 0.5 mg/kg and one exceed the acceptable concentration for Pb of 10 mg/kg) also proves a relatively good condition of the Polish cosmetics market and suggests insubstantial risk for the potential consumers. The results gathered for some of the eye shadows intended for children turned out to be alarmingly high, in particular for elements such as Cd. The highest concentration of Cd reached almost 4 mg/kg, while of Pb amounted to 16 mg/kg. The presence of the statistically significant differences was confirmed for all included parameters with an exception of the color of the eye shadow. Considering the results acquired only for Cd and Pb with respect to the country of origin, the least contaminated cosmetics by metallic impurities seem to be the one produced in Canada, while the ones presenting the highest health risk among all studied eye shadows are make-up cosmetics originating from Poland and Italy. Multivariate analysis of a large data set using CA methods and PCA provided valuable information on dependencies between variables and objects.

## 1. Introduction

For people around the world, the beauty make-up segment, color cosmetics in particular, plays a key role in their everyday life [1,2]. A cosmetic can be defined as ‘any article intended to be rubbed, poured, sprinkled or sprayed on, or introduced into, or otherwise applied to, the human body or any part thereof for cleansing, beautifying, promoting attractiveness, or altering the appearance, and includes any article intended for use as a component of cosmetic’ [3]. Frequently applied cosmetics are mixtures of some surfactants, oils and other ingredients (e.g., pigments), and by definition, consumers expect that they will be effective, long-lasting, stable and safe to humans. Among quite wide range of products used in a daily basis, we can distinguish lipstick and lip gloss (used to color the lips); powder and rouge (employed to color the face, light and remove flaws to produce an impression of health and youth); mascara (used to enhance the eye lashes), eye liner and eye shadow (used to color the eye lids); and nail polish (used to color the fingernails and toenails) [4,5]. According to the Environmental Working Group and based on the study called ‘Exposures Add Up’, the typical woman every day uses approximately 12 products that can contain, in total, about 168 unique ingredients. Moreover, the study revealed that when these products are used on a regular basis, 1 in 13 women can be exposed to components that are known or probable human carcinogens, while 1 in 24 women can be exposed to ingredients that are known or probable reproductive and developmental toxins, which are directly linked to impaired fertility or developmental harm for a baby in the womb or a child [6]. Along with the growing interest in our appearance there are also some concerns regarding the physiological and behavioral effects of trace metals in human tissues [1,4]. Naturally, cosmetics should undergo safety tests before they can be placed on the market involving the comparison against acceptable limits for, e.g., chosen elements [7]. Unfortunately, some reports suggest that cosmetics commonly used by women and children can contain toxic metals [1,8,9].

The risk of make-up cosmetics use is mostly associated with their frequent application, and a potential health hazard should be considered from this point of view. Even though the ingredients, chemical structures, toxicity profiles and exposure patterns are regularly controlled, some of the so-called side effects may occur in the form of immediate visible symptoms (e.g., caused by well-known allergic metals, such as Ni, Co or Cr, leading to local effects on human skin, such as sensitization or allergic/irritant contact dermatitis), while others may appear with the prolonged use of cosmetics (e.g., mineral particles with nano size). For example, if the cosmetics contain nanosized metal oxide pigments, special attention should not only be paid to the possible creation of metal ions but also the inevitable penetration of human body and subsequent accumulation into secondary organs. In the case of eyeshadows, the risk of developing irritative and/or allergic skin reactions via percutaneous absorption of the pigments and pigments impurities seems to be particularly serious since this group of color make-up products are applied in the peri-ocular area, where the facial skin is thinnest (0.55 mm, whereas, in other regions of the face, it could be even 2 mm) [8,10]. Moreover, due the fact that many toxic elements and their compounds are water soluble, moist skin can subsequently promote the absorption process [1]. It has been reported in the literature that heavy metals, such as Pb and Cd, are quite common impurities of pigments used, especially in lipsticks, eye shadows and face powers due to the fact that these products offer a wide variety of colors and shades. It was shown that those metals can be absorbed by children’s and women’s skin when using these cosmetic products [11]. What should be underlined is that children are more susceptible to heavy metal toxicity than adults and have greater exposure potential due to hand-to-mouth activity. Moreover, other factors such as the concentration of the component in the product, the amount of product applied, the site of application (e.g., lips vs. eyes), the length of time left on the skin and the presence of emollients and penetration enhancers in the cosmetic product should be included in the health hazards simulations. All these variables make the possible assessment of dermal absorption by single components very complex, especially for well-documented and performed dermal absorption studies, where all mentioned factors would be included, so they are still not completed [5]. Due to this fact, many countries have introduced their own limits for chosen elements (and compounds) since the assessment of heavy/toxic metal limits in cosmetics based on human health risk is such a huge challenge. In general, the presence of toxic metals in cosmetics products should be prohibited or at least restricted in the regulations of many countries [4]. The established restrictions and standards regarding the permissible level of various metals are inconsistent and different for various products and countries [12]. Additionally, during the last few decades, we have been witnessing the growing interest in the safety of cosmetic product studies. This type of research is gaining more attention and has developed rapidly since the 1960s when, at that moment, it was widely believed that cosmetic products are not capable of penetrating the surface of the human body [13]. Recently, the number of papers in which some elevated levels of various toxic elements are stated is increasing each year. A good example is the research conducted by Health Canada, which demonstrated a serious problem in cosmetic products safety testing. The survey was dedicated to 49 makeup products from various price ranges (from inexpensive brands to more expensive brands), and it included 14 eye shadows, 8 lipsticks or lip glosses, 7 mascaras, 5 foundations, 5 blushes or bronzers, 4 concealers, 4 powders and 2 eyeliners. It was found that all products were tested positive for heavy metals presence, while none of them was listed on the product’s label. In all of the products, the presence of nickel was confirmed, 96% contained lead and 90% contained beryllium. Among all studied samples, only in the case of one cosmetic product, the presence of four elements (As, Cd, Hg, Pb) recognized as the contaminants of the most concern was not confirmed (the results for these elements in this sample were below the detection limit). For the rest of the analyzed samples, the presence of at least one out of four of the mentioned elements was positively verified. The study also revealed that typical product tested in this work contained at least four out of eight elements of interest, namely As, Be, Cd, Hg, Ni, Pb, Se and Tl [6].

The brief literature review ([1,7,9,11,13,14,15,16,17,18,19,20,21,22,23,24,25,26] Table S1) concerning the evaluation of different elements in cosmetics products, including eye shadows, shows that the most popular technique being employed for this purpose is Atomic Absorption Spectrometry (both Flame AAS and Graphite Furnace AAS), followed by ICP-MS (Inductively Coupled Plasma-Mass Spectrometry) and ICP-OES (Inductively Coupled Plasma—Optical Emission Spectrometry). Techniques such as XRF-type (X-Ray Fluorescence), for which special sample preparation is not needed, are not so commonly used in the safety assessment of cosmetics products. Techniques such as ICP-based or AAS require special sample preparation step for the so-called wet chemical analysis, which involves the treatment of samples with different reagents or their mixtures (HNO_3_, HClO_4_, HF, HCl and H_2_O_2_). Most commonly, the concentrations measured are based on the amount of elements extracted from the base product due to the problems with the total decomposition of studied materials (the addition of HF is necessary in order to remove the, e.g., mica residues). Based on the data presented in Table S1, Supplementary Materials, four elements that are the most frequently determined are Cd, Cr, Ni and Pb. Countries in which the evaluation of the level of various elements is conducted cover many parts of the world and include, among others, Nigeria, Brazil, Italy, Saudi Arabia or Iran.

Eye shadows, in particular, being the eagerly used products by women and children, have been reported to contain toxic metals. They can be present in various forms, such as pencils, anhydrous creams, emulsions, sticks and pressed powder, and are highly pigmented makeup products. For all these reasons, eye shadows in this work were carefully chosen as a type of cosmetic products to evaluate the safety in reference to the level of seven metals determined by the ICP-MS technique (Ag, Ba, Bi, Cd, Pb, Sr, Tl) in a total of 94 samples widely available on the Polish market. The selection of samples was made in order to include several parameters important from the point of view of the typical consumer, such as product type (mat/pearl), consumer group (for adults and children), price range (very cheap, medium price, expensive and very expensive), color (12 different colors were tested), manufacturer (brand—products from 8 producers in total) or country of production (origin—products from 4 countries tested). Being aware that make-up products are also produced for children (a group particularly exposed to the toxic elements presence), for comparison purposes, products intended to be used by children over 3 years of age were included in the studied sample.

This study was aimed at assessing the safety of using the eye shadows commercially available on the Polish market based on the determined levels of some chosen elements. With regard to this goal, the obtained results were compared with the literature data and recommendations regulated by law in Poland. Moreover, the multivariate analysis was performed to study the potential relationship among the levels of tested metals in reference to considered factors (such as color or brand). The risk connected with the application of this type of beauty product was calculated based on the threshold values suggested in the literature and presented in the second part of this manuscript along with the results for other metals (Co, Cr, Cu, Hg, Mn, Ni).

## 2. Results and Discussion

Eye shadows typically contain ingredients such as: talc, mica, titanium oxide, zinc oxide, micronized silk powder, calcium or magnesium carbonate, magnesium or zinc stearate, rice powder, kaolin and pigment. The basic functions of the individual ingredients, along with their average content expressed in percentages, are summarized in Table S2, Supplementary Materials. Eye shadows are cosmetic products that are available in a wide range of colors due to the pigments present in their composition, such as iron oxide, titanium dioxide, copper powder and chromium oxide [27]. Some of the substances that are added to the eye shadows play a special role. For example, titanium dioxide guarantees an opaque effect, while bismuth oxychloride and micas provide a pearlescent effect [27,28,29]. Elements such as Ag, Al, Au or Cu have been reported to secure an iridescent metallic effect to the final product [15,27], whereas metals including Cd, Co, Ni or Pb are most probably accidental impurities left as a trace signature during the production of eye shadows in metallic devices or are raw material contamination, especially in pigments [27,28,29]. As already mentioned, the possible sources of studied elements in analyzed eye shadows products with regard to the official recommendations on cosmetics products are presented in Table S3, Supplementary Materials.

In this study, the concentrations of 7 metals in 94 samples of eye shadows were given, namely Ag, Ba, Bi, Cd, Pb, Sr and Tl. The majority of the studied materials was produced in Poland (*n* = 50) and in China (*n* = 26). Products manufactured in Poland were eye shadows mostly belonging to the cheapest price range (36 samples from the category “1” and 14 samples from the group “2”). Among all studied eye shadows, only products originating from Poland were classified exclusively to the less expensive cosmetics. Samples from Canada and Italy were grouped in the category of expensive “3” (ITA, *n* = 8) or very expensive “4” products (ITA, *n* = 3 and CAN, *n* = 7). Products from China were eye shadows covering a wide price range (groups “2”, “3” and “4”).

### 2.1. The Permissible Values

In 2018, the new regulation in Poland was introduced regarding cosmetic products (Dz.U. 2018 poz. 2227) [30]. This act defines the obligations of entities and the competence of authorities to perform their duties and administrative tasks resulting from another regulation at the international level—Regulation (EC) No 1223/2009 of the European Parliament and of the Council of 30 November 2009 on cosmetic products [31]. Most of the European Union countries adopted Regulation EC 1223/2009. Even after “Brexit”, all cosmetic products placed on the market in the United Kingdom (England, Wales, Scotland and Northern Ireland) intended for sale, or to be given away for free, in the course of a commercial activity must comply with the mentioned regulation. According to Annex II of this international act, in the list of substances prohibited in cosmetic products, the following components in relation to elements determined in this study are included: lead and its compounds, cadmium and its compounds, thallium and its compounds, strontium lactate, strontium nitrate, strontium polycarboxylate, barium salts (with the exception of barium sulfide under some specified conditions), and of barium sulfate, lakes, salts and pigments prepared from coloring agents when listed in an additional annex to this act. However, in one of the points of this regulation (Article 17), it is mentioned that the non-intended presence of a small quantity of a prohibited substance, stemming from impurities of natural or synthetic ingredients, the manufacturing process, storage, migration from packaging, which is technically unavoidable in good manufacturing practice, shall be permitted. For this reason, internal standards have been introduced within individual countries.

In Poland, there is an internal regulation that specifies the maximum allowed concentration of elements in cosmetic products [32]. Permissible concentrations are defined for only a few of them. The elements for which the maximum levels are established are as follows: lead (max. concentration of 10 mg/kg), arsenic (max. concentration of 2 mg/kg), cadmium (max. concentration of 0.5 mg/kg), mercury (max. concentration of 1 mg/kg with some exceptions) and nickel (max. concentration of 10 mg/kg). In this work, the authors will mainly refer to these limits, even though there are some other regulations that detail the maximum values [33,34,35]. What should be also underlined is the fact that among seven metals quantified in this paper, some limit values can be found for only two of them (Cd and Pb). This means that although the amounts detected for other metals can be significant, the acceptable levels for them are not included in any standards, and the obtained results can be purely compared with the literature data. Thus, in Table 1, the permissible values solely for Cd and Pb that should not be exceeded are presented and expressed in units used in cited literature [32,33,34,35,36].

#### 2.1.1. The General Data Analysis

**Figure 1:**
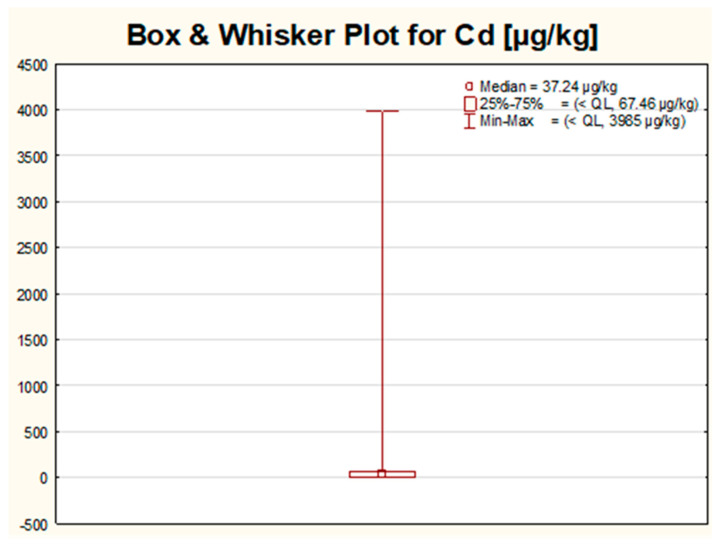
The box–whisker plots for Cd concentrations in the studied eye shadows (µg/kg).

**Figure 2:**
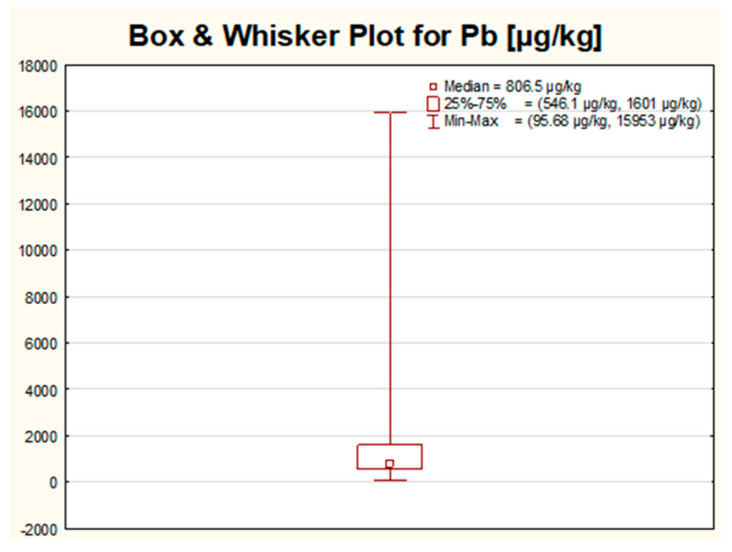
The box–whisker plots for Pb concentrations in studied eye shadows (µg/kg).

The created box–whisker charts for all of the seven analyzed variables have a very similar visage. For all metals, the greatest variation in the obtained results for all studied samples was observed within 25% of the highest values. Even though the same trend was noticed for all measured metals, for some elements, namely Ba, Bi and Cd, the diversity of the majority of the results seemed to be completely insignificant when compared with the length of the whisker, covering up the whole box. For the rest of the analyzed metals (Ag, Pb, Sr and Tl), the box was visible in the chart, but the width of the box was still slight in reference to the length of the whisker, with the median value directed towards the 25% of the lowest results (Pb, Sr, Tl) or located in the center of the box (for Ag). Two types of the aforementioned box–whisker plots for selected variables (Cd and Pb) are shown in Figure 1 and Figure 2 and used as an example of the general trend (a small number of the outliers compared to the total number of observations).

Based on the determined median values, the elements can be ranked into the following order: Ba » Sr > Pb > Bi > Ag > Tl > Cd (Table 2). Only for three elements in some samples, the presence of Ag, Cd and Tl was not confirmed. The results obtained for mentioned metals were, in general, at least one order of magnitude lower than for Ba, Sr, Pb or Bi. The highest range, variance and standard deviation were noticed for two elements: Ba and Sr, which may potentially suggest that these metals can be used for sample discrimination. The highest difference between mean and median values was stated for Bi (about a 50-times bigger mean when compared with the median value), followed by Ba (4.5-times higher) and Sr (2.5-times higher). Such a huge scattering, mainly in the case of Bi, implies that, for a limited number of samples, extremally high levels of Bi were determined, whereas, for the majority of the samples, the acquired concentrations were even a few orders of magnitude lower (the highest concentration of Bi quantified in this work reached a value of 1184 mg/kg, and it was an almost 4000-times higher value than the assessed median value for this metal). In contrast to Bi, the lowest variance of the results was observed for the concentration of Ag, Cd and Tl. These elements most probably will have the weakest discrimination power in terms of samples differentiation according to selected criteria, such as brand or country. This hypothesis was later confirmed by the multivariate analysis, where, e.g., for all these metals, the length of the vectors on the factor plane for the first two components was the shortest.

#### 2.1.2. The Lowest Results in This Study for All Studied Metals

As it was already underlined for Ag, Cd and Tl, some results were below the established limit of quantification. Silver was not detected in 22 out of 94 samples of eye shadows for adults. Fourteen samples (out of 22) in which the traces of Ag were indicated originated from China. Moreover, 16 out of 22 samples were produced by only two companies (six samples by C company, while ten by company A), and the majority of them were pearl eye shadows in, e.g., grey (light and dark) colors. It should be noted that all samples dedicated for kids were tested positive for silver. Cd was below the limit of quantification in the case of 34 samples. Fourteen samples were derived from Poland (samples from brands B, F, G and K), while the rest originated mostly from C company (seven samples) and A company (eight samples). The level of thallium was not quantified in 15 samples (4 out of 15 samples were produced by company C, while 3 by company G). Eight out of fifteen samples were manufactured in Poland. Most of these samples were pearl eyeshadows in green color (four samples) and in navy blue color (three samples). Any other resemblances were not found.

The presence of strontium, barium, lead and bismuth was confirmed in all studied samples. The lowest values of Sr were reported in samples produced by company A and K, while for the rest of the studied metals (Ba, Pb and Bi), the lowest levels were determined in samples mostly manufactured by company L (eye shadows for kids) and E (the only brand belonging to the group 4 in terms of the average product price of 1 g of product). All the indicated samples belonged or to the “2” price range (company A, K and L) or to the “4” prince range (the most expensive brand among all studied—E). The colors that were the most commonly noted for samples defined by the lowest trances of Sr, Ba, Pb and Bi were golden, copper-colored, beige or dark grey, and the type of product was mainly pearl, with the exception of brand L (products dedicated for kids).

#### 2.1.3. The Highest Results in This Study for Cd and Pb

The highest values for Cd were found for samples produced by companies F, G, L and C, mainly in Poland. The majority of these samples were the cheapest eye shadows from the group category named “1” in this study. The highest result for Cd among all the studied samples reaching 4 mg/kg was reported for pink eye shadow produced in Poland and dedicated for children. In the group of the highest ten results for Cd was another product from the same company (brand L) in green color, which may suggest the common source of Cd impurities. However, not all samples from company L demonstrated elevated traces of Cd, probably due to the fact that products manufactured by this company were produced in two different countries (Poland and China). The second highest result (0.6 mg/kg) was about four times lower and was achieved for dark grey eye shadow manufactured in Poland by company G. Both companies for which the highest values were reported, have their headquarters in Poland.

Based on the data gathered for Cd, it can be concluded that the obtained results are generally low and do not exceed the limit established and accepted in Poland (0.5 mg/kg), with the exception of two samples discussed above. The highest result acquired in this study exceeded almost eight times the regulatory value, while the second-highest result of 0.6 mg/kg was only slightly higher than the limit value. However, according to the most restricted permissible levels established in Germany (Table 1), for which the limit values for Cd should be not higher than 0.1 mg/kg, in our work, for 17 out of 94 tested eye shadows (amounting for about 18% of all samples) this value was exceeded.

Our results for Cd in terms of median, mean or maximum values were quite comparable with the results reported by other authors [9,13,14,17,18,19,20,23,24,25]. The values further given are expressed in units used in the cited literature. Obtained by Iwegbue et al., 2016 [17], the highest value of Cd of 5.10 µg/g was in the same order of magnitude as the one reached in our work. Quite similar findings were published by Abrar et al., 2018 [20], who reported the highest concentration of Cd at a level of 2.23 mg/kg. However, in other papers, e.g., by Kalicanin and Velimirovic, 2016 [18], and by Igwo-Ezikpe et al., 2017 [19], Cd was not detected in the studied samples. Much higher levels of Cd than in our work can be found in the study by Nourmoradi et al., 2013 [11], in which Cd levels ranged from 1.54 to 55.59 µg/g or in the work by Mousavi et al., 2013 [7], who notified Cd levels within the range from 0.52 to 1066 mg/kg.

In the case of Pb, the analysis of the ten highest results revealed that in most of the cases, the highest values of lead were stated for pearl-type eye shadows produced by Polish companies from the cheapest price shelve (group “1” or “2”). The colors of the eye shadows which appeared in this group most frequently were sea color followed by purple. The highest result obtained in this work for Pb (16 mg/kg) was stated for a pearl sea color eye shadow produced by company C in Italy from the expensive (group “3”) price shelf. This was the only result that exceeded the permissible level required in Poland for cosmetics and amounting to 10 mg/kg. Apart from this concentration, for two other samples, the concentrations of lead were also very close to the limit value and exceeded 9 mg/kg. Both pearl samples were produced in Poland (by companies B and G) and were from the cheapest (group “1”) price shelve in purple and dark grey colors. Again, when comparing the obtained results for Pb with the highly restricted limit value established in Germany and amounting to 2 mg/kg, then about 15 out of 94 measured eye shadows (approximately 16% of the total number of tested products) went beyond this permissible concentration. We can conclude that, according to Polish limits, only in two products, the level of Cd was elevated, and in one product, the concentration of Pb was overdrawn. When the limits from Germany were taken into account, then more or less one-fifth of the studied samples exceeded the limits for Cd and for Pb (in eight cases, the same samples were beyond the limit values acceptable in Germany for both Cd and Pb).

The mean value, median and the highest results for Pb in this work stayed in agreement with the concentration ranges proposed in the literature data. The values further given are expressed in units used in the cited literature. The most similar results to the ones obtained in our work for Pb were declared by Sainio et al., 2000 [15], who acquired Pb concentrations within a wide range from below the detection limits up to 16.8 ppm. Additionally, the levels of Pb quoted by Barros et al., 2015 [16], which varied in the range from 1.222 to 9.632 ng/mg, seemed to be relatively comparable. Quite similar values were proposed by Iwegbue et al., 2016 [17], who declared Pb concentrations within the range of 0.30–21.6 µg/g. Nevertheless, eye shadows products investigated by Kalicanin and Velimirovic, 2016 [18], had much higher values than in our work (Pb concentrations ranged from 26.43 up to as high as 95.55 μg/g). Even wider ranges of Pb concentrations were shown by Mousavi et al., 2013 [7], from 0.5986 to 202.058 mg/kg. A slightly lower range for Pb (from below the detection limit up to 45.859 ppm) was reported by Söğüt et al., 2016 [21]. On the contrary, one of the lowest values for Pb was presented by Lim et al., 2017 [22], who quantified the levels of Pb in quite a narrow range between 14.2 and 284.4 µg/kg, and by Ahmed et al., 2017 [23] (from 18.55 to 181.90 µg/kg).

Such a small number of samples exceeding the permissible values (two samples went beyond the limit value for Cd and one for Pb) proves a relatively good condition of the Polish cosmetics market. Quite worrying are some of the results acquired in products intended to be used by children. In the literature data, various scenarios regarding the cosmetics’ safety were described. For instance, Abrar et al., 2018 [20], investigated 25 samples of eye shadows of different colors purchased in Pakistan. The study revealed that 80% of the samples showed high Pb contents, while 26% of the samples showed Cd contents above the permissible limits. On the contrary, Lim et al., 2017 [22], analyzed forty samples of eye shadow, and in any of them, the lead concentrations exceeded the permitted levels. Similarly, in the paper published by Zainy et al., 2019 [25], Pb and Cd were absent in most of the studied samples. However, it should be kept in mind that in studies dedicated to eye shadow analysis, different decomposition procedures are applied. Based on the literature review included in Table S1, the following acid mixtures were employed and described in the literature to perform the wet chemical elemental measurement by spectroscopic methods: HNO_3_/HF/H_2_O_2_ [1,16], HNO_3_/HCl/H_2_O_2_ [17], HNO_3_/HCl [15,18], HClO_4_/HNO_3_ [19], HNO_3_/H_2_O_2_ [11,20,22], HNO_3_/H_2_O_2_/HF, HCl [23], HNO_3_/HF/HCl [24], HNO_3_/HClO_4_/H_2_O_2_ [7] or HNO_3_/HF [14]. The proportions between the mass of the sample being decomposed and the number of reagents used, along with some information regarding the cosmetics preparation step, are presented in more detail in Table S1. Some of the recommended procedures involved in the addition of HF may result in a total sample decomposition. In our study, the metals were extracted with the use of the mixture of HNO_3_ and H_2_O_2_ as proposed elsewhere [11,20,22]. Thus, the differences in the levels of metals between our results and the ones published may be a consequence of the dissimilarities in the applied procedures. On the other hand, the total sample decomposition does not seem to be required since the main risk is mostly connected with the extraction of eye shadow components under the influence of the sweat and their absorption via skin, and less by swallowing like in the case of the lipsticks.

#### 2.1.4. The Highest Results in This Study for Ag, Ba, Bi, Sr and Tl

The highest results obtained for the other five studied metals, although not included in any standards, were analyzed with the main purpose of finding some common characteristics.

In the case of Ag, no mutual quality could have been identified in terms of color, country of origin or price. The highest result for Ag reaching 1.3 mg/kg was determined for the pearl golden sample produced by company C in Italy. Moreover, it can be noticed that in all products for children, the content of Ag was successfully detected but at a very narrow range of concentration between 0.1 mg/kg to 0.3 mg/kg. Surprisingly, Ag presence was also confirmed in all matte eye shadows (with one exception).

Our results for Ag stay within limits published in the literature. The values further given are expressed in units used in the cited literature. The presence of Ag was not confirmed in the eye shadows in work by Söğüt et al., 2016 [21]. Farrag et al., 2015 [1], reported Ag concentrations in the eye shadow ranging from <1.704 ppm to 2.217 ppm. Similar results were given by Issa et al., 2016 [13], who obtained Ag levels from 0.157 up to 2.183 mg/kg. The highest values of Ag were described by Zainy et al., 2019 [25]. In this work the determined silver concentrations ranged from below the detection limit up to 137.62 mg/kg.

The majority of samples within the ten highest results for Ba were produced in Poland (9 out of 10) mostly by companies B or G and were from the cheapest price range. Many of them were in pink color. The highest individual result for Ba and reaching as high as 2154 mg/kg was measured in Polish eye shadow of green color and manufactured by company F. In 11 out of 12 results for brand F, in 10 out of 12 for brand G and in 9 out of 12 cases for brand B, the concentration of Ba was above the 15 mg/kg. All mentioned products were produced exclusively in Poland, and elevated concentrations of Ba seem to be typical for our country.

The results published for Ba stay in agreement with our data. The values further given are expressed in units used in the cited literature. Söğüt et al., 2016 [21] declared the concentrations of Ba in the range starting from below the detection limit up to 878.230 ppm. Slightly lower values for Ba were suggested by Issa et al., 2016 [13], where the Ba levels ranged from 21.30 to 66.15 mg/kg, and Zainy et al., 2019 [25], where the Ba concentrations were within the range from 52.18 mg/kg to 267 mg/kg. Much higher maximum values of Ba than in our work, reaching as high as 4724.62 ppm, were given by Farrag et al., 2015 [1].

The results for Tl were strongly positively correlated with the levels obtained for lead. Samples containing the highest levels of Tl were pearl products of sea or grey color manufactured in Poland or in Italy. The two highest individual results for Tl, which were above the value of 0.6 mg/kg, were quantified in products of grey color (light and dark) produced by the same company G, from Poland. For other samples from this company, only in three cases, the presence of Tl was not detected.

The highest results for Bi were stated in pearl eye shadows produced mostly by companies A and C in China or Italy. For 7 out of 12 samples produced by company A from China, the content of Bi was above 1 mg/kg, while for 11 out of 12 samples, the concentration of Bi exceeded 0.3 mg/kg. For brand C, only three samples had levels of Bi higher than 1 mg/kg, and in half of them, the concentration of Bi exceeded 0.3 mg/kg, with the majority of samples originating from Italy. The highest individual result for Bi reaching 1184 mg/kg was detected for the mentioned company with production in Italy for pearl eye shadows of sea color. In the majority of matte eye shadows (12 out of 19), the content of Bi was below 0.3 mg/kg.

For about 8 out of the 94 tested eyeshadows, the level of Sr exceeded 10 mg/kg, from which as many as 7 originated from Poland. The highest concentration of Sr was recorded for the sample of navy blue color purchased from brand F and amounted to 63 mg/kg. The concentrations of Sr for other eye shadows from the same brand were also elevated when compared with the results for the rest of the studied samples. The opposite situation was stated for brand A. Samples manufactured by this company were characterized by relatively low amounts of Sr than other brands.

Only single reports can be found in the literature for Tl, Bi and Sr levels in eye shadows, and the values further given are expressed in units used in the cited literature. In the work by Farrag et al., 2015 [1], Tl levels were quite high when compared to our study and within the range of 4.133 ppm to 7.956 ppm. In the case of Bi, the published data suggest much lower values of Bi in eye shadows. Both median and mean values were within the limits given in the literature, but reported by other authors, the maximum values were far lower than the ones obtained by us. In the work by Söğüt et al., 2016 [21], Bi concentrations ranged from below the detection limit up to 1.186 ppm. Farrag et al., 2015 [1], obtained Bi levels in the range from 19.87 ppm to 97.36 ppm. Quite similar results were presented by Santos et al., 2018 [26], who detected Bi only in 2 out of 23 samples and maximum notified value for Bi was as high as 139 µg/g. The maximum Sr concentration determined by Farrag et al., 2015 [1], reached the value of 110.628 ppm and was about two-times higher than the highest value of Sr measured in our study; however, the mean and median values stayed in the ranges reported for this metal by these authors.

#### 2.1.5. The Summary of the Quantitative Data

The detailed analysis of the highest results acquired in our work for each metal proved that the same samples can be characterized by the substantially high values of more than one metal, suggesting that the level of some impurities can be correlated, creating the specific elemental fingerprint of eye shadow materials. Exemplary, two pearl eye shadow samples produced by company F in Poland from the cheapest price range (group “1”) of sea color and navy color can be defined respectively by the quite high concentration of Ag, Cd, Pb, Sr, Tl and Ba, Cd, Sr, Pb. Two pearl eye shadows produced by company G in Poland from the cheapest price shelve (group “1”) of dark and light grey colors can be both characterized by a high level of Ba, Cd, Pb and Tl. The two cheapest (group “1”) pearl samples manufactured in Poland by brand B of a purple color can be defined by the high concentrations of Ba, Cd, Pb and Sr, while of yellow color by simultaneously high levels of Ag, Ba, Pb and Tl. All these examples refer to samples produced in Poland from the cheapest price range.

It can be summarized that the results generated for all studied metals stayed within the limits proposed elsewhere. For all metals, the highest concentrations were comparable or lower than the maximum levels found in the literature. The only exception was Bi, which is not so commonly determined metal and for which only a few reference data can be found.

The average tested product in this study contained at least four out of seven elements of interest, namely Ba, Bi, Pb and Sr. The majority of them contained over 15 mg/kg of Ba (57 out of 94 samples), over 0.3 mg/kg of Bi (48 out of 94 samples), over 0.7 mg/kg of Pb (61 out of 94) and over 1.5 mg/kg of Sr (50 out of 94 samples).

### 2.2. Statistical Analysis

In this study, the influence of six different parameters was investigated. The samples were divided into groups, as described in the experimental section, and the differences in the concentration of studied groups were verified by statistical tests.

#### 2.2.1. The Influence of the Color

In the case of eye shadow color, any statistically significant differences were observed. This can be attributed to the fact that, in this work, no results for elements that can be associated with the color were presented. This parameter will be discussed in more detail in the second part of this study. Even though no statistically important differences were confirmed, a few general conclusions can still be presented. The highest individual results for Bi, Pb and Sr were noted in eye shadow of sea color; for Ag, the highest result was achieved for eye shadow of gold color; for Cd, the highest level was recorded for pink eye shadow; for Ba, the highest result was reported for eye shadow of navy blue color, while for Tl, the highest concentrations were measured for eye shadow of dark and light grey. Moreover, it can be stated that the highest values within 50% of the most typical values were acknowledged:

- for Ag in eye shadows of navy blue, copper and sea color;
- for Ba in eye shadow of pink color;
- for Bi in eye shadows of light grey, brown and sea color;
- for Cd in eye shadows of green, pink and light grey color;
- for Pb in eye shadows of sea and purple color;
- for Sr in eye shadows of purple, pink and sea color;
- for Tl in eye shadow of sea color.

Based on the gathered information, it can be stated that colors of eye shadows that are the most affected by the metallic impurities are most commonly pink, grey or sea color.

#### 2.2.2. The Influence of the Type

In terms of eye shadow type, the statistically significant differences in the concentrations of studied variables were confirmed only for Cd levels. Although matte-type samples were a minority (*n* = 19), higher individual maximum values along with bigger diversity of the most typical values and higher median and mean were noticed in matte group for Cd results. In refence to the mean values the Cd concentrations in matte eye shadows were about six-times higher, while based on the median values the results in the same group were about twice higher in contrast to the pearl eye shadows Different trend was observed for the rest of studied metals, for which higher individual results were recorded in the group of pearl eye shadows represented by 75 samples. The 50% of the most typical values seemed to be quite comparable in both groups. Higher median and mean values were proven in pearl eye shadows for elements such as Ba, Bi, Pb and Tl. Slightly higher mean values and approximately twice higher median values in matte eye shadows were noticed for Ag and Sr concentrations. The biggest differences between the central values for two studied groups were shown for Bi levels (about 60-times higher mean in pearl samples group, and about three-times higher median in pearl samples group).

#### 2.2.3. The Influence of the User

Another considered parameter was the user type, for which two types of samples were distinguished. The products intended to be used by children amounted to only 10 out of 94 studied samples. The statistically significant differences in the concentration of Ag, Ba, Bi, Cd and Pb were proven for this parameter. Only for Cd, the highest mean (about ten times higher), median (about two times higher) and the highest individual results were reported in group of eye shadows designed for kids use. Fort he rest of mentioned metals much higher median and mean values and higher individual results were stated in the eye shadows indented to be used by the adults. About twice higher mean and median values were determined in eye shadows for kids for metals such as Ag, Ba and Pb, while much more significant differences were proven between both studied groups of eye shadows for Bi concentrations (mean value in eye shadows for adults was 160 times higher, whereas median value was about 6 times higher). No differences were positively verified for Sr or Tl, for which slightly higher both median and mean values were proven in the eye shadows dedicated for the adults. 

#### 2.2.4. The Influence of the Price

The impact of the price of the product on the obtained results was checked as well. The products were divided into four groups in terms of their market value, where the cheapest eye shadows belonged to the “1” group, while the most expensive to “4”. The statistical analysis showed that the differences could be observed only for the concentration of the following metals: Ba (between groups “1” and “2” and between groups “1” and “4”), Bi (between groups “1” and “4” and between groups “2” and “4”), Pb (between groups “1” and “2” and between groups “1” and “4”) and Sr (between groups “1” and “2” and between groups “1” and “3” and between groups “1” and “4”). For all these metals, the differences in their levels for the same pair (between the cheapest eye shadows “1” and the most expensive “4” ones) were confirmed. In all cases, group “1” was characterized by the higher variation of the whole population and a much higher median, unlike group “4”. It should also be underlined that for Bi, much higher individual values were quantified in group “3”, and the highest variation of the results was reported within group “2”, while for Pb, the highest individual values were observed in group “3”, but this time the biggest scattering of the results within the whole data set was seen within group “1”, followed by group “2”.

**Figure 3:**
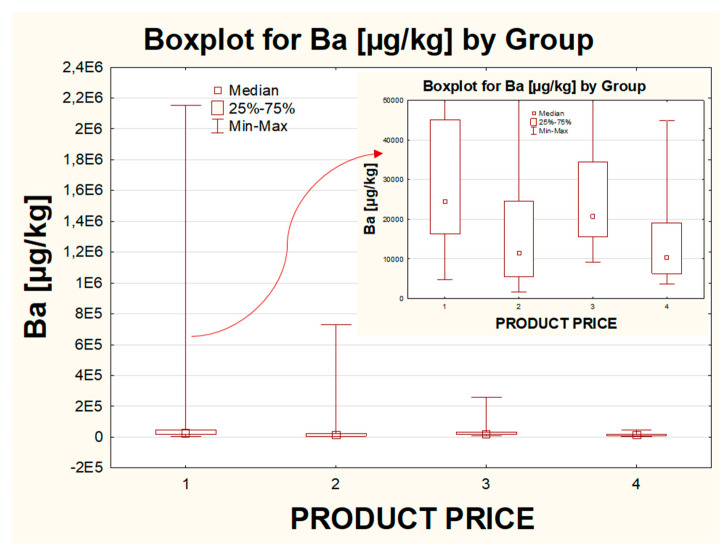
The box–whisker plot representing the influence of the product price on the concentration of Ba (µg/kg), where the cheapest products—group “1”, medium price products—group “2”, expensive—group “3” and very expensive—group “4”.

Some interesting trend was noticed for elements such as Ag, Ba, Bi, Cd, Pb and Tl, for which, in general, the lowest median value, the smallest variation of the most typical values and the lowest diversity of the results within the whole population as well as the lowest individual values were reported for the group with the most expensive eye shadows (group “4” represented exclusively by the brand E). Additionally, the highest individual values were stated in group “1” (eye shadows from the cheapest price shelve) only for Ba, Sr and Tl. The elements that seem to represent the possible influence of the price on their concentration are Ba (Figure 3) and Sr, for which the highest variability of the most typical values and the highest median value were reported within group “1”, in contrast to the lowest variability for most typical values obtained for group “4”. In the case of the results obtained for the groups “2” and “3”, no clear trend can be found in the results for Ba and Sr. The decreasing trend in the levels of the studied metals, along with the increase in the product price, can be demonstrated based on the values of the outliers; however, they should not be treated as a good representation of any tendency since they do not reflect the central values.

#### 2.2.5. The Influence of the Country of Origin

**Figure 4:**
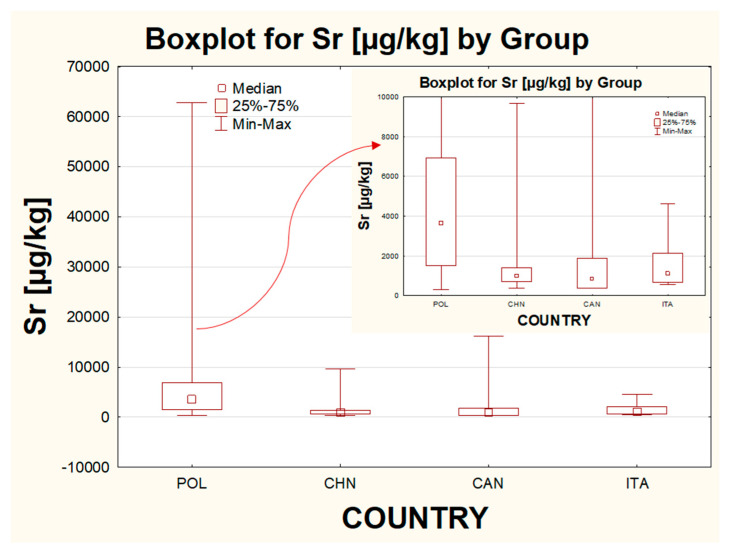
The box–whisker plot representing the influence of the country of origin on the concentration of Sr (µg/kg), where POL—Poland, CHIN—China, CAN—Canada, ITA—Italy.

Another parameter taken into account was the country of origin. Four different groups were distinguished, with the majority of samples coming from Poland (*n* = 50) and China (*n* = 26). Within the studied category, the statistically significant differences in the concentration were revealed for Sr and Ba (between POL and CHIN), for Pb (between POL and CHIN and between POL and CAN) and for Bi (between CHIN and CAN and between POL and CAN). For metals such as Ba, Cd and Sr, the highest median value, the highest individual results and the biggest variance within the whole population were stated for samples originating from Poland. The lowest median value among all of the studied groups was observed for products from Canada for elements such as Bi, Cd, Pb and Sr. For Sr, the median value for Canadian products was only slightly lower than for Chinese eye shadows. For metals such as Ag, Ba and Tl, the lowest median was reported for samples from China. For metals such as Ag, Ba, Bi, Cd, Pb and Sr, the highest median value was recorded in the group of Polish eye shadows. For Bi, the median value for the group of cosmetics from Poland was only a little bit higher than for the group of Chinese eye shadows. The highest results within considered groups for Ag, Bi and Pb were indicated for eye shadows from Italy, while for the rest of the analyzed metals (Ba, Cd, Sr and Tl), the highest concentrations were determined in samples from Poland. Taking into account all the gathered information, some general conclusions can be drawn. Based on the data acquired for metals included in the Polish standards and investigated in this work, the least contaminated eye shadows seem to be ones produced in Canada, while the ones presenting the highest health risk are eye shadows from Poland and Italy. However, samples from Poland were represented by the widest range of samples, whereas samples from Italy were (along with products from Canada) the minority. The exemplary influence of the country on the concentration of Sr is presented in Figure 4.

#### 2.2.6. The Influence of the Brand

**Figure 5:**
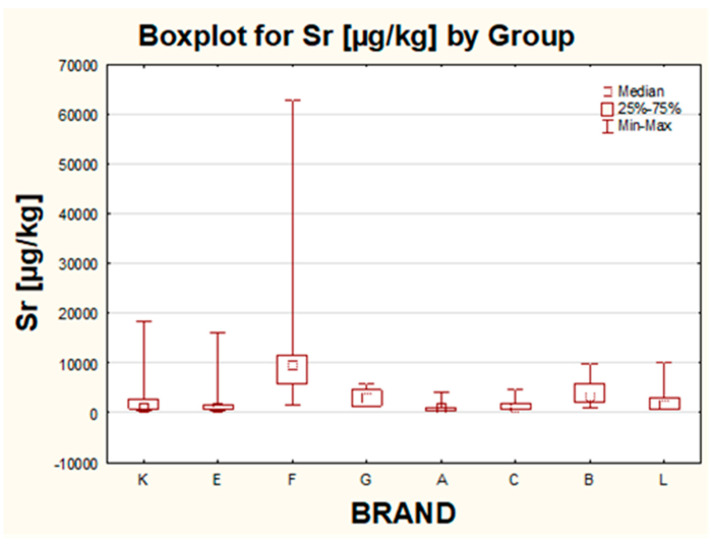
The box–whisker plots representing the influence of the company on the concentrations of Sr (µg/kg).

The last parameter included in this work was the influence of the brand. In total, eight different companies were studied. Moreover, within one brand, in some cases, the production was carried out in a few countries (for brand C, in CHIN and ITA, for brand E, in CHIN, CAN and ITA and for brand L, in CHIN and POL). The only brands represented exclusively by one country were A, B, F, G and K, out of which brand A was from China, and B, F, G and K originated from Poland. Statistically significant differences in the concentration of analyzed metals were proven for Ag, Bi, Cd, Pb and Sr. For Ba and Tl, no influence of the company on the obtained results was found. For Sr, the differences in the levels of this metal were observed for the following pairs: K–F, E–F, F–A, F–C and A–B. The highest median value and the highest individual results for Sr were quantified for samples from Polish brand F. These samples can be possibly differentiated from make-up cosmetics from other brands based on elevated (when compared with other eye shadows in this study) Sr levels (Figure 5). The opposite situation was noticed for products manufactured by brand A, for which within the whole population, the lowest median value and lowest scattering of results were observed. Products from both companies were produced in only one country. It can be suggested that maybe the Sr concentration can be used as a discrimination factor for the company’s influence for products manufactured in Poland.

**Figure 6:**
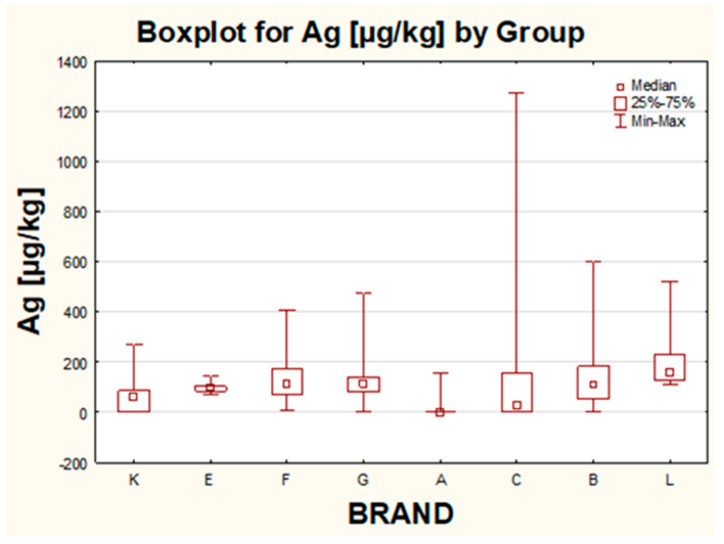
The box–whisker plots representing the influence of the company on the concentrations of Ag (µg/kg).

For Ag, statistically significant differences in the levels of this metal were proven between the following pairs K–L, F–A, G–A, A–B, A–L and C–L. For this metal, the highest median was reported for brand L (products dedicated for kids), and products belonging to this group were in general characterized by relatively high levels of silver, although the highest results for Ag were determined for brand C (Figure 6). Moreover, it can be noticed that within brand E the lowest diversity of the whole population was observed while the lowest 50% diversity of the most typical values and the lowest median were both shown for brand A.

In the case of Cd, the statistically significant differences in the concentrations of this metal were distinguished between pairs: E and L; A and B; A and L. The highest median values of Cd were shown for company B and L, but for brand L, the highest individual results were gained as well, which substantially influenced the attained statistically significant differences between the studied pairs in reference to the brand. The lowest median values were observed for brands A and C.

The highest number of statistically significant differences was affirmed for Bi, for which they can be specified by the following pairs: K–E, K–L, E–F, E–A, F–L, G–L, A–B and A–L. For Bi, the highest median and the highest scattering within 50% of the most typical values were shown for brand A, but all the comparisons for this metal were definitely strongly affected by the highest individual results acquired for brands K, A and C., while the lowest results for Bi were generated for brands E and L.

A relatively similar scenario was shown for Pb, for which the differences in lead levels were confirmed for the following pairs: E–F, E–G, E–B, F–L, G–L and B–L. The highest median value was indicated for brand B. However, substantially high individual results were acquired for a few brands, namely K, F, G, C, B or L, creating crucial data scattering, which had a direct impact on the generated differences in the results between the mentioned pairs. The lowest dispersion of the results, being all at a relatively low level, was presented by company E, while the lowest median values were detected both for company L, followed by brand E.

All things considered, the pairs of brands that appeared in the presented comparisons the most frequently were A–B and A–L. With the exception of Bi, for Ag, Cd and Sr, brand B was always defined by a much higher median value, much higher individual results and much higher scattering within the whole population in contrast to brand A. The same trend was noticed also for other studied elements (Ba, Pb and Tl), even though no statistically important differences in their concentrations were verified positively. In the case of the other pair (A–L), for Ag and Cd, but not for Bi, brand L was also characterized by a much higher median value, higher individual results and bigger variance within the whole group than samples from brand A.

To summarize, it can be pointed out that products from Chinese company A seem to be the least affected by the metallic impurities determined in this work, which stays in agreement with already drawn conclusions. On the contrary, one of the highest results in this study for the analyzed metals was recognized for products from Polish companies (F and G) and products manufactured in Italy (brand C).

#### 2.2.7. Summary of the Parameters Studied

Taking into account the influence of all the factors considered in this study, we can state that, for Bi and Pb, the impact of the same parameters (brand, country, price and user) was shown. The Tl concentration has demonstrated the influence of any factor on its concentrations. The only metal for which the influence of the type of product was positively checked was Cd. The parameters most frequently listed were: user (Ag, Ba, Bi, Cd and Pb), brand (for Ag, Bi, Cd, Pb and Sr), price (Ba, Bi, Pb and Sr) and country of origin (Ba, Bi, Pb and Sr). The most critical factors discussed in this study appear to be the user type, the country of origin and the price of the product. In the case of the brand, in general, the statistical comparisons were highly affected by the highest individual results acquired within the studied groups.

The influence of various factors was also discussed by other authors. The values further given are expressed in units used in the cited literature. Abrar et al., 2018 [20], investigated eye shadows locally available in Pakistan. In their work, in twenty-five samples of five colors (red, golden, orange, white and pink), the level of Pb, Cd, Cr and Zn was analyzed. Higher concentrations of Pb in pink and white colors than in other colors were confirmed. Moreover, these colors were also characterized by higher concentrations of Cd. Mousavi et al., 2013 [7], determined the content of Pb and Cd in 120 samples of 15 brands of pressed powder eye shadows of seven colors: pink, white, violet, brown, golden, green and blue produced in different countries and purchased in Iran. The authors reported that the brown and golden colors in all studied brands had the highest concentrations of lead, while blue and green colors were characterized by the lowest. In the case of cadmium, the golden and blue colors showed the highest and lowest concentrations of this metal, respectively. Moreover, the highest concentration of lead and cadmium was detected in Chinese eye shadows when compared with samples originating from Iran, Korea, England and Turkey. Ahmed et al., 2017 [23], quantified the levels of Pb, Cd, As and Hg in 21 popular international eye shadow brands from China, France, Italy and Ireland, which were widely available in Saudi Arabia. Eye shadows in this work were categorized into three different types (cheap, medium and expensive) based on their price. The authors reported that the concentration of cadmium was relatively high in cheap eye shadow samples compared to medium and expensive products, while the lead concentration was higher in expensive eye shadows samples than in medium and cheap samples. The highest individual results for Pb and Cd were obtained in eye shadows from France and China, respectively. Barros et al., 2015 [16], determined the level of Pb in eye shadow and blush samples for adults (sixty) and for children (twenty-four) of different brands and colors. The study confirmed similar levels of Pb in eye shadows for children and adults. However, Pb levels in blushes for children turned out to be higher than in the blushes for adults. Kalicanin and Velimirovic, 2016 [18], in their work, analyzed 10 eye shadows samples available in the Serbian market, among other beauty products. Cd presence was not confirmed in the studied samples, whereas Pb concentrations were higher in pearl-shine eye shadows compared to the eye shadows without this addition. In another study by Omolaoye et al., 2010 [9], eye shadows imported from China of 20 colors from seven brands collected in Nigeria were analyzed to determine the levels of heavy metals: Pb, Cd, Ni, Cu, Zn, Cr, Co and Mn. The lowest concentration for lead and cadmium stated in this work were below the detection limit: in three colors for Pb (army green, brown, purple) and thirteen colors for Cd (white, black, pink, army green, purple, light yellow, light brown, golden brown, ash, in two brown colors). Only for two brands (of three different colors, namely pink and blue, both by the brand called Diamond, and light yellow by Midle), the lead content was higher than 20 µg/g. Seven out of twenty colors (35%) of the brands of eye shadows contained cadmium at low concentrations, but the highest individual results above the 4 µg/g were determined in eyeshadows of a dark grey color by Ladyhood, in pink by Diamond and in blue by FBI brands. A similar study was performed by Zainy, 2019 [25], who quantified in 70 samples from Saudi Arabia and Egypt (39 cheap blushers and 31 cheap eye shadow samples) belonging to different brands the concentration of the following metals: Pb, Mn, Cd, Ag, Au, Cu, Cr, Ni, Ba, Fe, Al, Zn and Ti, with respect to their colors. The sources of these products, which were all at a cheap price, were KSA, USA, Turkey, China and Egypt. Ag was detected in only one sample of eye shadow of a blue color. The highest results for Ba were reported for white, gold, green and violet eye shadows. Cd was quantified in only three eye shadows: two of violet color and one of green color. Two significantly higher concentrations of Pb were obtained in two violet eye shadows. Nourmoradi et al., 2013 [11], investigated the content of lead and cadmium in the most frequently used brands of cosmetic products (lipstick and eye shadow) in Iran. The eye shadows products consisted of three colors by five brands. A significant difference between the average lead content in the different brands of eye shadows was stated. The statistical analysis has also not confirmed a meaningful difference between cadmium content for various brands of eye shadows. Moreover, the authors observed that eye shadows with a golden color had a higher concentration of cadmium, but there was no significant difference between lead content of golden color eye shadow vs. other various colors. The influence of the color of eye shadows on the content of Cr and Pb was also studied by Lim et al., 2017 [22], who collected forty samples and divided them into five color categories: pinks, blues, greens, browns and grays. For each color category, eight popular local and imported brands of eye shadows in Malaysia were analyzed. The results showed that lead concentrations were found to be the highest in the blue color category. The presented examples prove that the most frequently studied factor is the color of eye shadows. Although the authors define the samples in their research quite well, it is not so common in the literature to notice some more references with regard to the country of origin or the brand. It is also not so typical to analyze more than one–two factors at once. Moreover, in many of the published papers, the results acquired for products being sold outside of Europe are mostly discussed, where eye shadows samples are quite often mixed with other beauty cosmetics products.

### 2.3. Multivariate Analysis of Studied Eyeshadows

A tree diagram was made for all of the tested samples. In the obtained graph, a grouping of objects into two branches was observed; however, it was difficult to say unequivocally which features of the objects decided about the grouping. The division into two tree branches is typical for Ward’s method. Therefore, in the next stage, the additional charts were prepared, where the objects were coded as those representing the considered parameters, e.g., the product’s country of origin (Figure S1, Supplementary Materials), the producer (Figure S2, Supplementary Materials) or the price (Figure S3, Supplementary Materials). On the basis of the analysis of all graphs, it can be seen that Ward’s method divided the samples in such a way that the left part of the tree diagram includes eye shadows produced mainly in China by company A, and these products are from a relatively low-price range—group “2”). The analysis of the remaining clusters within the second branch allows us to draw another conclusion that the grouping of objects around the central part of the diagram is also closely related to both the product’s country of origin and the price since most of the eye shadows in that region were materials produced in Poland and from the cheapest price (group “1”). The same diagrams were made for the rest of the studied factors, but no univocal conclusions were drawn. In the case of the remaining factors, i.e., the division of shadows into products intended for children and adults, no clear trend was observed. Baby products represented by the 10 out of the 94 samples were relatively scattered between the center and the right side of the plot. The shadow colors, on the other hand, did not appear to be stacked in any particular groups; however, within this study, the results for metals that could be associated with specific colors, such as Cu, were not presented. In contrast, the matte shadows constituting 19 samples out of 94 were mostly clustered on the right side of the graph, with some minor exceptions.

The analysis by the CA method was also performed for the studied variables (Figure S4, Supplementary Materials). The prepared tree diagram represented the correlations between the determined levels of metals in tested eye shadows. It was shown that two independent groups of metals could be distinguished. To the first branch, the elements with heavy masses, such as Bi, Pb and Tl, belonged. The second group consisted of metals such as Cd, Ag, Ba and Sr. Moreover, it can be concluded that the pairs of metals that seem to present the highest correlation between their levels in measured samples are: Bi & Pb and Ba & Sr. The mentioned pairs of metals concentrations do not seem to be related to each other since their position in the tree diagram is located in separate tree diagram arms. This may suggest that the source of those two groups of metals is completely different.

**Figure 7:**
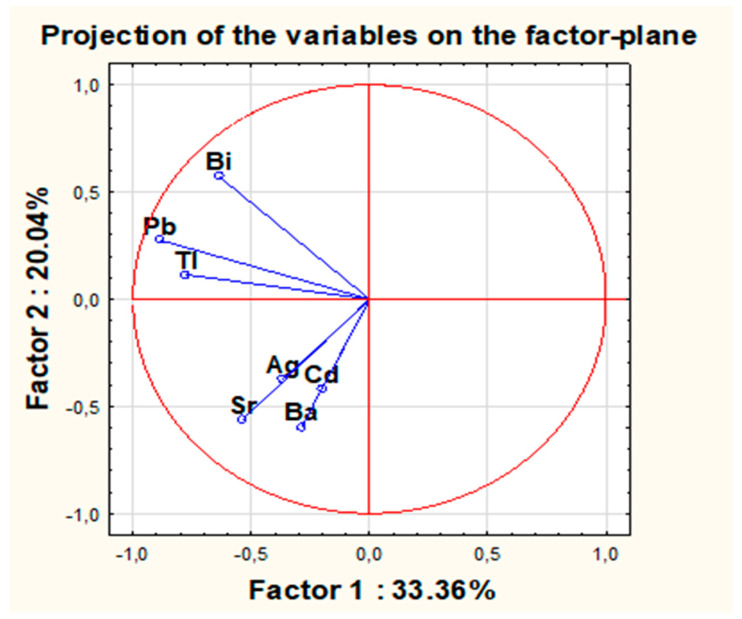
Projection of variables on the factor plane for the first two components.

The next chart (Figure 7) shows the projection of variables onto the plane for the first two principal components. It contains vectors connected to the origin of the coordinate system, which is represented by the original variables and placed on the plane defined by the first two principal components.

The analysis of the cumulative percent of variance suggests that the first two components account for only 53.4% of the total variance, and the first three components account for 66.9% of the variance. As many as the first five components explain more than 89% of the total variance. However, eigenvalues exceeding the value of 1 apply only to the first two components. For the next two components, the eigenvalues exceed 0.88 but not the value 1. The analysis of the chart also suggests that all variables are negatively correlated with the first component, while only four variables (Ag, Sr, Cd, Ba) are negatively correlated with the second component. The most strongly correlated variables with the first component are: Pb (−0.88), Tl (−0.78) and Bi (−0.64), while with the second component: Ba (−0.60), Sr (−0.56) and Bi (0.57). The weakest influence on factor 1 is shown by Cd (−0.20) and Ba (−0.29), while on factor 2—Tl (0.11) and Pb (0.27). The position of the vectors in relation to each other also indicates that the following groups of variables can be distinguished: strongly correlated variables, such as Cd & Ba, as well as Ag & Sr (very close proximity of four vectors within this group with similar length of vectors for Sr and Ba and slightly shorter comparable vectors of Ag and Cd), as well as Tl and Pb and Bi (vectors represented by the primary variables of Tl and Pb are located much closer to each other than for Bi, but still, they all lie in one quadrant). There seems to be no correlation between the specific variables, for example, between Bi and Sr (Ag) and Bi, as well as between Pb with Ba (Cd), due to the perpendicular position of the vectors in relation to each other. Variables given in parentheses have much shorter vectors; hence, their impact on individual components seems to be much smaller than the other original variables.

**Figure 8:**
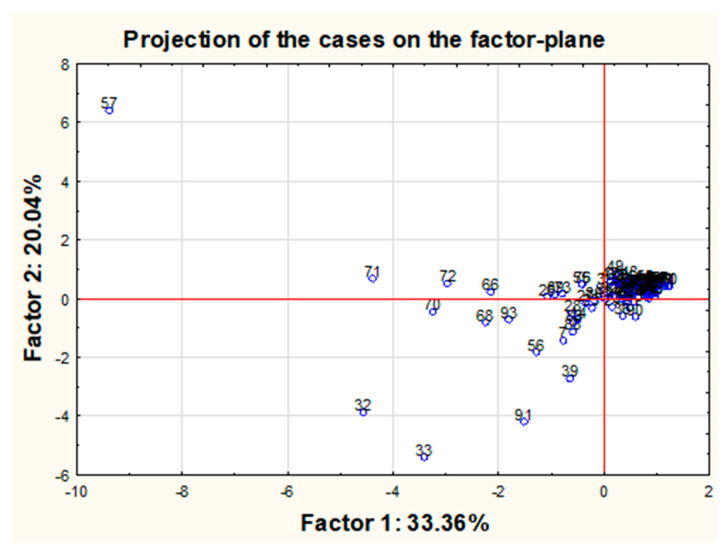
Projection of objects for the first two components for all studied eye shadow samples after the scale change.

Moreover, the PCA analysis was used to graphically present projections of the original cases onto the plane formed by the first two components (Figure 8).

**Figure 9:**
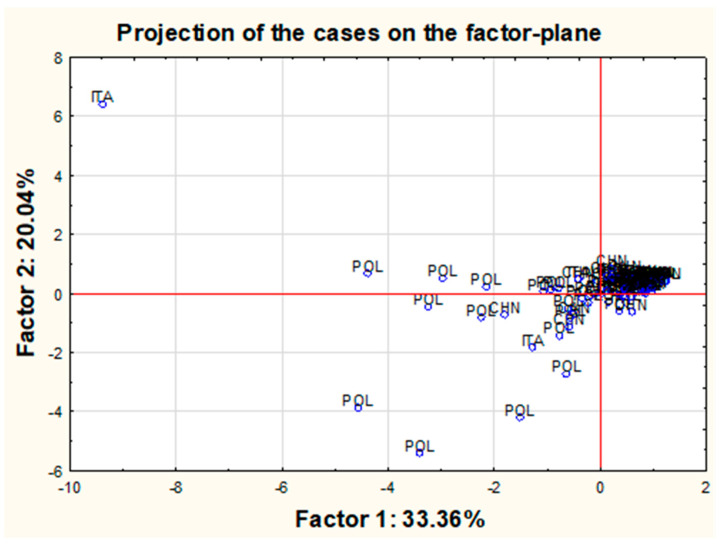
Projection of objects for the first two components for all studied eye shadow samples after the scale change in relation to the country of origin.

The analysis of the chart suggests that most of the points are gathered around the central cluster. However, it is possible to list a few outliers, in particular, object no. 57, clearly separated from the rest of the objects and several other objects deviated much less from the central cluster, such as objects no. 32 and 33 or 70, 71, 72 or 91 or 39 and 56. Additionally, it was shown that all these samples are characterized by the maximum concentrations obtained for one or more of the tested variables and higher than the mean value of the other variables. For example, sample No. 57, as the biggest outlier from the rest of the objects (pearl shade produced by the company C in Italy in a sea color from a relatively high price shelf—“3”), has very high concentrations of Sr, Ba and Tl and the highest concentrations of Pb and Bi among all of the tested samples. Sample No. 32 (pearl shade produced by the company F in Poland in a sea color from the cheapest price range—“1”) has the highest concentrations of Sr and a very high concentration of Pb, while sample 33 (pearl shade produced by the company F in Poland in a navy blue color with cheapest price shelf—“1”) can be characterized by very high concentrations of Sr and Pb and the highest level of Ba out of all the studied eye shadows. Sample 70 (pearl shade produced by the company K in Poland in a sea color from the mid-range price range—“2”) has very high concentrations of Sr, Ba and Pb. Sample 71 (pearl shade produced by the company G in Poland in a dark gray color from the cheapest price range—“1”) and sample 72 (pearl shade produced by the G company in Poland in a gray color from the cheapest price range—“1”) both have very high concentrations of both Ba and Pb. Sample 91 (matte shade produced by the L company in Poland in a pink color from the mid-range price range—“2”), which is intended for use by children, can be defined by relatively high concentrations of Ba and Sr and the highest concentration of Cd. Sample 39 (pearl shade produced by the company G in Poland in a green color from the cheapest price shelf—“1”) has the second-highest concentration of Ba, while sample 56 (pearl shade produced by the company C in Italy in a gold color from a relatively high price shelf—“3”) has a high concentration of Ba and the highest concentration of Ag among the other tested objects. Sample 66 (pearl shade produced by the company C in Italy in a copper color from a relatively high price shelf—“3”) has a very high concentration of Ba and Sr. Sample 68 (pearl shade produced by the company B in Poland in a sea color from the cheapest shelf price—“1”) has a high Ba concentration, while sample 93 (matte shade produced by the L company in China in a purple color from the relatively cheap price shelf—“2”) has a lot of Ag and Sr. In all the examples quoted, it can be seen that the indicated samples are characterized by concentrations of some elements that, compared to other concentrations, could be considered as the outliers. At the same time, it should be noted that this method is extremely sensitive to outliers, and perhaps removing them from the data set would increase the percentage of explained variance. However, hasty exclusion of data points (especially outliers) is not always justifiable since it can strongly interfere with the data set, and statistical patterns may consequently differ. The analysis of outliers itself suggests some general conclusions. Considering the concentrations of all outliers, it is possible to identify a “general profile” of samples with these deviating values: in most cases, these results correspond to samples in shades of blue (blue, sea color or violet), they are mainly pearly eye shadows from the cheapest or relatively cheap price range, and in the overwhelming majority, they are manufactured in Poland (by companies G and F) (Figure 9). Two samples were found to differ from the rest (sample 93 and 91) related to products intended for children in pink and violet colors.

**Figure 10:**
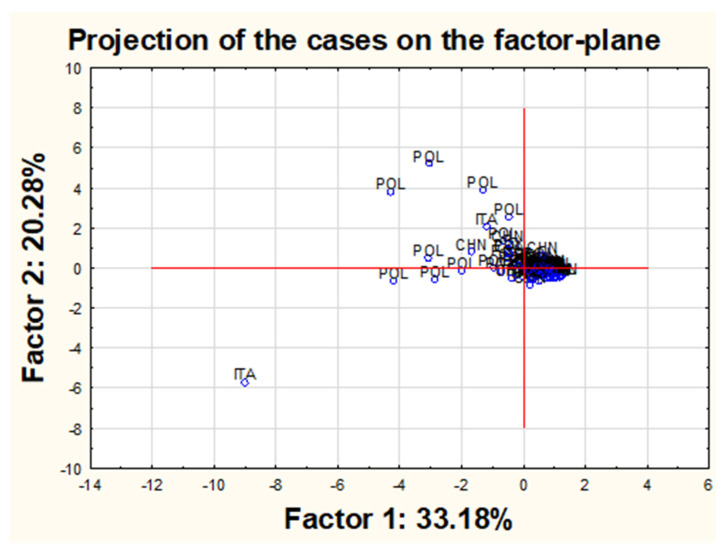
Projection of objects for the first two components for reduced data set (after the rejection of the following outliers: samples No. 32, 33, 39, 56, 57, 66, 68, 70, 71, 72, 91 and 93).

A final attempt was made to verify if any possible changes may occur in the PCA analysis after the listed above-mentioned 12 outliers (samples No. 32, 33, 39, 56, 57, 66, 68, 70, 71, 72, 91, 93) would be removed from the data set. For a new reduced group of samples, renewed PCA analysis was performed (Figure 10). The explained variation for the first two components almost has not changed. Moreover, new “outliners” have been revealed. Most of the samples located at some distance from the central cluster can be described in general as samples produced in Poland, belonging to the “1” or “2” price shelf and in general in sea, pink or violet color. The presented profile is quite similar to the one already proposed. Additionally, the inference among the variables has not changed, and the projection of variables for the first two components was nearly identical to the one before the data set reduction.

Due to the failed assumption of the normal distribution of the analyzed variables, and in order to assess the correlation between the concentrations of studied metals, the so-called Spearman’s rank coefficients were tested. In this case, rank correlation coefficients measure the statistical dependence (degree of correlation) between rankings of two variables in such a way that the values of the variables have been replaced with ranks assigned inside the interval from −1 to 1. Based on the analysis of the Spearman’s rank order correlation results, it can be concluded that there are statistically significant correlations between the ranks for the following pairs of variables: Sr–Ag (r = 0.49); Sr–Cd (r = 0.46); Sr–Ba (r = 0.57); Sr–Tl (r = 0.37); Sr–Pb (r = 0.67); Ag–Cd (r = 0.69); Ag–Pb (r = 0.23); Cd–Ba (r = 0.22); Cd–Pb (r = 0.34); Ba–Tl (r = 0.56); Ba–Pb (r = 0.71); Tl–Pb (r = 0.60); Pb–Bi (r = 0.34). Thus, a positive correlation included in the category of very high correlation (ranks between 0.7 and 0.9) can be observed for the relationship between the concentrations of the pairs of the following variables: Sr–Pb, Ag–Cd and Ba–Pb. A positive correlation classified to the high correlation category (r between 0.5 and 0.7) can be found for the relationship between the concentrations of the pairs of variables: Sr–Ba, Sr–Pb, Sr–Ba, Ba–Tl and Ba–Pb. The strongest negative correlation of Spearman’s rank was found for the relationship between the concentrations of pairs of variables: Ag–Bi (r = −0.13), but it was not statistically significant. The observed strong correlations for some particular pairs of metals can be directly linked with their common source. For example, Ba and Sr can be present in the same colorants, as shown in Table S3, Supplementary Materials. The obtained conclusions correspond to the information obtained through the analysis using the CA and PCA methods for the determined levels of metallic impurities.

## 3. Materials and Methods

### 3.1. Samples

This work, compared to other works in this field, stands out in terms of a large number of samples tested, a large number of determined elements and a wide range of factors simultaneously taken into account. Quite commonly, eye shadows are tested as one of many groups of beauty products (lipsticks, blushes, eyeliners, mascaras, etc.). This highly limits the number of tested samples of eye shadows and has a significant impact on obtained results that may not be representative in relation to the local market. Moreover, in most of the papers, only one element is analyzed (e.g., Pb) or from two to four (typically Pb, Cd, Ni, Cr). The determination of levels of more than four elements is very rare. Elements for which the levels are not so often given are Ba, Bi, Hg, Sr or Tl. Regularly, the authors include one or max. two factors at once (such as the country of production, color or brand) and very exceptionally divide samples into subgroups of consumers (e.g., for kids and adults). This paper is one of two parts dedicated to eye shadows analysis. The obtained results were grouped into the techniques used and the properties of metals studied. In the first part, the data gathered for toxic elements were presented, while in the second part of our survey mostly results for allergic elements were discussed. The results included in the first part were quantified by the ICP-MS technique, while all the results summarized in the second part were established by AAS-based techniques (FAAS—Co, Cr, Cu, Mn, Ni—and CVAAS—Hg). Thus, in this study, the elemental composition of seven metals (Ag, Ba, Bi, Cd, Hg, Pb, Sr and Tl) in a wide range of samples (94 eye shadows), which were divided into classes (consumer group, color, product type, country, brand and price range) was investigated. All attention was focused on assessing the impact of the six mentioned factors on the levels of measured elements. In the Supplementary Materials, some specific information about the studied elements is given in Table S3 in reference to their possible source in the cosmetic products and with regard to the legal records.

A total of 94 eye shadows samples were collected for the tests of this research. The studied cosmetics originated from four countries: Poland, Italy, China and Canada. The research focused on the analysis of 12 selected colors of eye shadows (yellow, brown, light grey, beige, pink, sea green, navy blue, gold, copper, purple, dark grey and green) with the aim to cover as widely as possible the huge variety of colors of products available on the Polish market. For this reason, within one company, in addition to the eye shadows palettes, single eyeshadows were also examined. As a consequence, in some cases, one brand was represented by more than one country since the production under one company name could be carried out in different places. Thus, for company A, the production was only held in China; for company B, F, G and K, the production was only in Poland; for brand C, the eyeshadows were manufactured in China and Italy; E—in China, Italy and Canada; brand L—in China and Poland. Moreover, brands were grouped into categories based on the price range established for a 1 g of product. To summarize, samples were analyzed according to the following criteria:

- product intended user (two groups): products dedicated for children (*n* = 10; one producer, ten different colors: yellow, light grey, beige, pink, sea green, navy blue, gold, copper, purple and green) and adults (*n* = 84; twelve different colors from seven different manufacturers: yellow, brown, light grey, beige, pink, sea green, navy blue, gold, copper, purple, dark grey and green);
- colors (12 groups): yellow (*n* = 8), brown (*n* = 7), light grey (*n* = 8), beige (*n* = 8), pink (*n* = 8), sea green (*n* = 8), navy blue (*n* = 8), gold (*n* = 8), copper (*n* = 8), purple (*n* = 8), dark grey (*n* = 7) and green (*n* = 8);
- type of product (two groups): matte (*n* = 19) and pearl shades (*n* = 75);
- country of origin (four groups): China (*n* = 26; company A, C, E and L), Poland (*n* = 50; company B, F, G, K and L), Italy (*n* = 11; company E and C), Canada (*n* = 7; company E);
- brands (eight groups): A (*n* = 12; China), B (*n* = 12; Poland), C (*n* = 12; China (*n* = 4), Italy (*n* = 8)); E (*n* = 12; Italy (*n* = 3), China (*n* = 2), Canada (*n* = 7)), F (*n* = 12; Poland), G (*n* = 12; Poland), K (*n* = 12; Poland), L (*n* = 10; China (*n* = 8), Poland (*n* = 2));
- price range (four groups) based on the price of products calculated against one gram of product, from which the following groups were distinguished: the cheapest products (group “1”, *n* = 36, to which brands B, F and G belong), medium price products (group “2”, *n* = 34, to which brands A, K and L belonged), expensive (group “3”, *n* = 12, represented exclusively by brand C) and very expensive (group “4”, *n* = 12, represented only by brand E).

### 3.2. Samples Preparation and Equipment

#### ICP-ToF-MS

Wet chemical analysis by the ICP-MS technique requires a prior preparation step, which includes samples mineralization. For this purpose, 4 mL of 65% HNO_3_ (Baker) and 1 mL of 30% H_2_O_2_ (Sigma-Aldrich) were added to a weighed mass of the sample (about 0.1 ÷ 0.2 g). The samples prepared in this way were introduced into the UltraWAVE system (Ultrawave system, Milestone, Via Fatebenefratelli, Italy). The digestion process of the studied material was carried out in accordance with the selected program for sample decomposition for mineral matrices, such as fly ash. The selected program was also applied for the tested CRMs and blank material and can be characterized by the quite restricted conditions (in terms of the program length or the maintained temperature), as described below in detail. Since no HF acid was added, the authors decided to use a program that will support the sample extraction in some critical conditions (such as for the fly ashes matrix that can be linked somehow with a mineral matrix of, e.g., mica present in eye shadows). The mineralization process consisted of two steps:

- Stage I (25 min): max. pressure inside the reactor 120 bar, max. temperature inside the reactor 250 °C with a microwave power of 1500 W (the power is adjusted in such a way that, at the end of stage I, the temperature of 250 °C will be reached inside the reactor);
- Stage II (10 min): max. pressure inside the reactor 120 bar, max. temperature inside the reactor 250 °C with a microwave power of 1500 W (in this stage, the power will be adjusted that the temperature of 250 °C will be sustained during this step).

Along with the studied samples, certificate reference material was measured of NCS ZC81002b Human Hair (China National Analysis Center for Iron&Steel, NCS Testing Technology Co., Ltd., Beijing, China), and satisfactory agreement was achieved (Table S4, Supplementary Materials) for elements listed in the certificate (Ag, Ba, Cd, Pb, Sr). Additionally, the authors have chosen one eye shadows sample that was decomposed and prepared in the same way as the studied sample and was analyzed by both techniques employed (FAAS and ICP-MS). The results were compared and used as an indicator for further analysis. Collaterally, after the sample decomposition step, the same eye shadow sample was additionally spiked with Tl (Merck, Darmstadt, Germany) and Bi standard (Merck, Darmstadt, Germany) in such a way that the final concentration of these elements in the sample was 60 µg/kg. In the same way, a separate sample of certified reference material of human hair dopped with Bi and Tl was prepared after the process of its wet decomposition. The concentration of Bi and Tl was determined both in the sample of the certificate reference material of human hair and in the selected sample of eye shadows (treated as the laboratory reference material) before and after the addition of the Bi and Tl standards. The measured Bi and Tl concentrations in sample of the certificate reference material of human hair and in the selected sample of eye shadows without the spiking (of Bi and Tl) were at negligible levels when compared with the amount of standard added. The average recovery values obtained by the ICP-MS technique for Bi and Tl (elements not included in the NCS ZC81002b Human Hair certificate) and for other determined in this study metals in the eye shadow matrix and in the spiked human hair certificate reference material were above 90% (Table S4, Supplementary Materials). Described eye shadows were analyzed during each working day as a reference with a matrix common for studied material by ICP-MS technique. Moreover, after every fifteenth sample, the standard containing studied elements at a concentration of about 10 µg/kg was analyzed by ICP mass spectrometer.

All the materials were extracted in the same way. After the mineralization process, the contents of the tubes were quantitatively transferred to class “A” flasks and filled with distilled water up to a volume of 25 mL. The tested eye shadow samples were not completely mineralized due to the presence of aluminosilicates in them (hydrofluoric acid was not added to the reaction mixture); hence, the entire mineralization stage of these samples was treated as more of an extraction of the selected elements than the total decomposition. The exclusion of HF from the decomposition mixture consequently forced the removal of solid residues not decomposed in the mineralization process via filtration with a pressure pump system.

Before the quantitative analysis by the ICP-MS technique, it was necessary to optimize the parameters of the mass spectrometer. For this purpose, a similar optimization procedure was performed as described by Pawlaczyk et al., 2019 [37]. The parameters and measurement conditions of ICP-ToF-MS (OptiMass 8000, by GBC, Melbourne, Australia) was given in the work by Gajek et al., 2021 [38].

For the quantitative measurement, it was necessary to prepare the calibration curve based on a standard solution of Merck VI (Multi-element ICP standard, Merck, Darmstadt, Germany) with an initial concentration of 1000 mg/kg. The calibration curve included the following standards made after the proper dilution of the base solution: 0 µg/kg; 2.0 µg/kg; 5.0 µg/kg; 10.0 µg/kg; 20.0 µg/kg; 50.0 µg/kg; 100.0 µg/kg; 200.0 µg/kg. On the basis of the blank samples prepared in the same way as the tested material (the same proportions of concentrated nitric acid and hydrogen peroxide were used), which also included the sample decomposition step supported by the microwave energy, the limits of quantification were also determined for each of the element being quantified (Table 3). For each decomposition series (consisting of 15 samples in total), the blank sample was made. Every sample and every blank were measured in three replicates. In total, 7 blanks were analyzed, and the mean value and the SD were assessed for the studied elements. The limits of quantification for the whole analytical procedure were calculated according to a formula: mean value + 6·SD.

The precision in each concentration level was high and have not excided 5%. The lowest precision was observed in the range of concentrations below the QL and was reaching < 10%. For high concentrations exceeding the calibration range (e.g., individual results for Ba or Bi), samples were doubly checked by diluting them to assess the exact concentration.

### 3.3. Data Analysis

The STATISTICA 12.5 (New York, NY, USA) software was used for statistical and multivariate analysis. The normality of distribution was assessed by Kolmogorov–Smirnov and Shapiro–Wilk tests. Due to the lack of the normal distribution of the studied variables (the concentrations of 7 metals), the Kruskal–Wallis non-parametric test was employed to assess the significance of differences in the determined levels of metals among particular groups according to the parameters considered, such as consumer group, type of product, brand, color, country of origin as well as price. The quantitative data were expressed in this study as box and whisker plots with a median value chosen as a central value. This type of diagram was used to show the differences in the set of samples within the analyzed variables as well within the considered groups in relation to the selected parameters, such as brand or product price. The box was represented by 50% of the most typical values results, whilst the whiskers were limited by the lowest and highest results obtained in this study. The greater variation of the most typical values of a studied variable was reflected by the wider box.

Additionally, multivariate analyses, including principal component analysis (PCA) and cluster analysis (CA), were applied, aiming to increase the interpretability of the results.

Cluster analysis is based on multivariate data analysis, which includes the idea of dividing objects into clusters (groups). Membership to a group is determined by the similarity of the objects established on the basis of the similarity matrix. In order to ensure a balanced influence of all variables (objects) on the elements on the similarity matrix, the data were subjected to an appropriate standardization process (the mean was zero and the variance was equal to 1) depending on the type of diagram which being created (for objects or for variables). In the hierarchical method, clusters are formed starting from the smallest ones (clusters inside the similarity matrix are searched, where the larger numerical values in the similarity matrix suggest differentiation of objects, while lower—the similarity). The choice of the Ward method as an amalgamation (linkage) rule was made as a method of combining objects or variables successively into those of increasing distance. The Ward method is considered to be universal and is based on the variance analysis (sum of the squared deviations of individual objects or variables between the centroid and the points of the new cluster being formed by linking both clusters is determined). As a distance measure, the squared Euclidean distance was chosen to give more weight to objects (variables) that are further away.

The idea of the PCA method is based on the determination of completely new variables (the so-called principal components), which are a linear combination of primary variables. Through the principal components analysis, it becomes possible to identify the variables that have the greatest impact on the appearance of individual principal components. Subsequent components should be mutually orthogonal (uncorrelated), and their extraction is related to the rotation that maximizes the variability (variance) of the original variable space while minimizing the variability (variance) around this variable. The number of components is equal to or smaller than the number of original variables, and each of them will explain only a part of the variability of original variables. On the basis of the eigenvalues, conclusions can be drawn about the part of the total variability that is “translated” by the principal component. The first of the principal components will always explain the largest part of the variance, and the subsequent ones will explain the portion of the variance that has not yet been explained by the previous components. Thus, the total variance will be the sum of the eigenvalues of each of the components and will refer to the share of individual eigenvalues, while the percentage of variation defined by it can be calculated. The coordinates of the end of the vector tell us how big the influence of the variable is on the given principal component (how much this variable contributes to the explanation of the variance of a given component). The longer the vector, the greater the contribution of a given variable to the structure of a particular component (the closer the vector is to the edge of the circle, the more this variable is represented by specific principal components). The quarter in which the vectors are located will also provide information about the type of correlation (positive or negative). In turn, the angle between vectors informs about the degree of correlation between individual original variables. Generally speaking, the smaller the angle, the stronger the relationship between the variables, which vectors are situated next to each other. If the vectors are positioned at 90°, then the correlation between the variables is supposed to be weak. The closer the angle between vectors of individual variables is to 180°, the more negative correlation can be observed [39].

## 4. Conclusions

Based on the determined median values for the studied metallic impurities by ICP-MS technique after the wet chemical analysis, their levels can be ranked into the following order: Ba » Sr > Pb > Bi > Ag > Tl > Cd. Only three elements were not present in all studied eye shadows, namely Ag, Cd and Tl. Silver was not detected in 22 of the 94 samples of eye shadows. Cd was below the limit of quantification in the case of 34 samples. The level of thallium was not positively quantified in 15 samples. The results obtained for the mentioned metals were about one order of magnitude lower than for other determined metals, such as Ba, Sr, Pb or Bi. Considering all gathered results, it can be concluded that they are generally low, within the limits presented in the literature, even for the outliers. Moreover, the results do not exceed the limits established and accepted in Poland for Cd and Pb, with three exceptions. In the case of Cd, the highest result acquired in this work reached almost 4 mg/kg, which was about eight-times higher than the regulatory value, while the second-highest result of 0.6 mg/kg was only slightly higher than the limit value. For Pb, only in one product, the concentration of Pb was overdrawn (amounting to 16 mg/kg) when compared with the permissible limit for this metal. However, it should be underlined that the results obtained for some of the samples intended for children turned out to be alarmingly high, particularly for elements such as Cd. It can be generally concluded that the average tested product in this study contained at least four out of seven of the elements of interest, namely Ba, Bi, Pb and Sr.

With an exception of the color of eye shadows, for the rest of the studied parameters (brand, price, country of origin, type or user), the statistically significant differences were confirmed. Only for Tl concentrations, the influence of any of the considered factors was positively verified. For Bi and Pb, the impact of the same parameters (brand, country, price and user) was shown. In this study, the most critical categories included seem to be the user type, the country of origin and the price of the product. The producer parameter appeared to be highly influenced by the country of production and stated statistically significant differences dominated by the outliers. The performed PCA analysis was very helpful in the identification of “outliers”. The general profile of samples with the highest deviating values can be related to samples in shades of blue (blue, sea color or violet), mostly pearl-type from the cheapest (“1) or relatively cheap price range (“2), and in the overwhelming majority, they were manufactured in Poland (by companies G and F).

## Figures and Tables

**Table 1:** Comparison of the permissible content of Pb and Cd (mg/kg) in cosmetics being in force in the USA, Canada, Germany and Poland [35,36].

Metal	USA	India	Canada	Germany	Poland
**Cd**	not indicated	not indicated	<3 mg/kg	<0.1 mg/kg	<0.5 mg/kg
**Pb**	<10 mg/kg (<20 mg/kg for color products)	<20 mg/kg	<10 mg/kg	<2 mg/kg	<10 mg/kg

**Table 2:** The basic descriptive statistics for studied variables in all analyzed eye shadow samples (µg/kg).

**Elements**	**(µg/kg)**	**N**	**Mean**	**Median**	**Min**	**Max**	**Range**	**Variance**	**Std.Dev.**
Ag		94	123.8	93.58	<QL	1271	1271	$2.8\times 10^{4}$	168.0
Ba			82,247	18,061	1602	2,153,693	2,152,091	$8\times 10^{10}$	282,543
Bi			14,755	303.6	25.68	1,184,575	1,184,550	$1.5\times 10^{10}$	122,339
Cd			100.2	37.24	<QL	3985	3985	$1.7\times 10^{5}$	414.9
Pb			1543	806.5	95.68	15,953	15,858	$5.1\times 10^{6}$	2249
Sr			4119	1600	301.0	62,837	62,536	$5.7\times 10^{7}$	7528
Tl			106.9	72.44	<QL	672	671	$1.5\times 10^{4}$	123.2

<Quantification Limit.

**Table 3:** Determined limits of quantification (LOQ) (µg/kg) by the ICP-ToF-MS technique for the studied elements.

Element	LOQ (µg/kg)	SD (µg/kg)	Selected Isotope
Ag	0.29	0.02	^107^Ag
Ba	0.19	0.01	^137^Ba
Bi	0.13	0.01	^209^Bi
Cd	0.11	0.01	^111^Cd
Pb	0.25	0.02	^208^Pb
Sr	0.35	0.03	^88^Sr
Tl	0.13	0.01	^205^Tl

## Data Availability

Not applicable.

## Supplementary Materials

The following are available online. Figure S1: Tree diagram for all studied samples with regard to the country of origin of eye shadows, where ITA—Italy, CHIN—China, POL—Poland, CAN—Canada; Figure S2: Tree diagram for all studied samples with regard to the producer of eye shadows. In total 8 companies were distinguished: A (China), B (Poland), C (China and Italy); E (China, Italy and Canada), F (Poland), G (Poland), K (Poland), L (China and Poland); Figure S3: Tree diagram for all studied samples with regard to the price range of eye shadows, where: group “1”—the cheapest products (brands B, F and G), group “2”—medium price products (brands A, K and L), group “3”—expensive (brand C) and group “4”—very expensive (brand E); Figure S4: Tree diagram for all studied variables (metallic impurities) in all eye shadows samples: Table S1: The literature review regarding the determination of chosen elements (total content) in eye shadows products. The results are only given for elements investigated in our study (the data for other elements as well for other products than eye shadows were omitted) and expressed in units used in cited literature; Table S2: Characteristics of the main ingredients of eye shadow; Table S3: Source and application of metallic impurities in eye shadows products; Table S4: Results gathered for the certificate reference material dopped with Tl and Bi standards in such an amount that the final concentration in the studied sample was 60 µg/kg (the data for Bi and Tl are not given in the certificate of the NCS ZC81002b Human Hair).

## Sample Availability

Samples are available from the authors.
