# Supplementary material for: Determination of Metallic Impurities by ICP-MS Technique in Eyeshadows Purchased in Poland. Part I
Source: Molecules. 2021 Nov 8;26(21):6753. doi: 10.3390/molecules26216753 (PMC8587163; doi:10.3390/molecules26216753)
Supplement: Supplementary file 1 [file molecules-26-06753-s001.zip › molecules-1380356-supplementary.pdf]

Literature review

Table S1. The literature review regarding the determination of chosen elements (total content) in eye shadows products. The results are only given for elements investigated in our study (the data for other elements as well for other products than eye shadows were omitted) and expressed in units used in cited literature [1,7,9,11,13-26].

Samples	Elements	Technique of analysis	Year	Country	Sample preparation step	Results	Remarks	Reference
**Eye shadows** and blushes samples (60 for adults and 24 samples for children) of different brands and colors	**Pb**	**HR-CS GF AAS** (Pb) and **LS GF AAS** (Pb)	2015	Brazil	Direct solid sampling for HR-CS GF AAS. In the case of LS GF AAS 0.20 g of sample was mineralized in in a closed-vessel microwave-assisted acid-digestion system (3 mL of concentrated nitric acid, 2 mL of hydrofluoric acid and 1 mL of 30% (m/m) hydrogen peroxide). The digests were transferred to 50 mL Teflon tubes and heated in a block digester at 150 °C for 4 h to eliminate the remaining hydrofluoric acid	**Pb** concentrations in the eye shadows varied in the range from 1.222 to 9.632 ng/mg	The levels of Pb found in makeup for children and adult eye shadows were close. The results obtained by the two methods were in agreement at a 95% confidence level.	Barros et al., 2015 [16]
Samples of different brands of facial cosmetics (n = 160) including lipsticks, lip glosses/lip balms, face powders, **eye shadows**, eyeliners, eye pencils, blushes (rouges), mascaras. The cosmetic samples were popular brands,	**Cd**, **Pb**, Ni, Cr, Cu, Co, Zn, Fe and Mn	**FAAS**	2016	Nigeria	1.0 g of sample was placed into a Teflon vessel and treated with 20 mL of concentrated nitric acid, 10 mL of hydrochloric acid and 5 mL of hydrogen peroxide. Samples were heated to 125 ◦C for 2 h and finally made up to 25 mL with 0.25 mol/L HNO_3	**Cd** mean 4.70 ± 0.50 μg/g (median 4.5 μg/g) range 3.70−5.10 μg/g<br>**Pb** mean 15.3 ± 7.30 μg/g (median 17.7 μg/g) range 0.30−21.6 μg/g	The systemic exposure dosage (SED) values for these metals obtained from the use of these facial cosmetic products were below their respective provisional tolerable daily intake (PTDI)/or recommended daily intake (RDI) values.	Iwegbue et al., 2016 [17]

some of which were produced locally and others imported. Most of the imported products examined were from the USA, China, Korea, India, France, Italy, Taiwan and the United Kingdom.								
Twenty-five different samples of cosmetic beauty products (7 lipsticks, 5 lip glosses, 10 **eye shadows**, and 3 henna hair dyes) of different colors and produced by different manufactures, obtained from local shops	**Pb**, **Cd**, Zn	**PSA**	2016	Serbia	Samples of 2–3 g were treated with a mixture of concentrated nitric and hydrochloric acids, respectively 1:1.5 (V:V) and heated at a temperature of 60–80 °C for the duration of 2 h. Samples were filled up to a volume of 50 mL	**Pb** in eye shadows ranged 26.43 to 95.55 μg/g (pearl shine eye shadows had greater lead content (from 53.19 to 95.55 μg/g) compared to the eye shadows without this addition (from 26.43 to 50.41 μg/g)<br>**Cd** was not detected in the samples of cosmetic beauty products analyzed in this study	All eye shadow samples tested in this study can be potentially toxic.	Kalicanin and Velimirovic, 2016 [18]
Six different types of facial cosmetics makeup from different brands with a sample size of 30 each: powder (PW), **eye shadow** (ES), eye brow pencil (EB), lip stick (LS) and lip gloss (LG) samples. All	**Cd**, Cr, **Pb**, Ni	**FAAS**	2017	Nigeria	To one gram of sample concentrated perchloric acid ($HClO_4$; 10 mL) was then added, placed on a hot plate in fume hood and digestion was started at 50 – 60 o C for about two hours with intermittent addition of $HClO_4$ acid to prevent the mixture from drying out. 10 mL nitric acid (HNO_3) added to the	**Cd** not detected in eye shadows.<br>**Pb** the highest concentration was 1.025 ± 0.000 mg/kg with lowest < DL	Some of the facial cosmetic make ups used in Lagos, Nigeria contained heavy metals and high concentration of PAHs. It was observed that ES, EB and LS had the highest Pb concentration.	Igwo-Ezikpe et al., 2017 [19]

the products were imported into Nigeria (USA, Ghana, China, but eye shadows only originated form USA and China).					perchloric acid digest in the beaker. Digestion was thereafter continued for further 1 hour. The digest was transferred to 25 mL			
**Eye shadows** purchased from local market in Lahore (red, golden, orange, white and pink), the most commonly used colours were selected and total 25 samples were analysed	**Pb**, **Cd**, Cr, Zn	**GFAAS**	2018	Pakistan	One gram of each sample was digested with 5 mL of concentrated nitric acid on a hot plate at 80 °C until the sample was dried, then the process was repeated again by adding 5 mL of concentrated nitric acid and dried on a hot plate at 80 °C. Then 1mL of concentrated hydrogen peroxide (H_2O_2) was added. The residual material was diluted to the final volume of 50 mL	**Pb** mean concentration ranged from 7.56-15.33 mg/g **Cd** mean concentration ranged from m 0.41- 2.23 mg/g	80% samples showed high Pb contents while 26% samples showed Cd contents above the permissible limits.	Abrar et al., 2018 [20]
Various brands in products of cosmetic products ranging from blush, **eye shadow**, lipstick, nail polish, shampoo, hand cream, cellulite cream, baby powder, soap, to toothpaste (50 samples)	V, Cr, Mn, Ni, Cu, As, Br, Mo, **Ag**, Sn, I, Ba, Hg, **Pb** and **Bi**	**XRFS**	2016	Turkey	Dried samples were homogenized using a mixer. Samples were pressed into the pellets.	**Ba** ranged from < Dl to 878.230 ppm **Pb** ranged from < DL to 45.859 ppm **Bi** ranged from < DL to 1.186 ppm **Ag** not determined in eye shadows	Pb levels in two out of 6 measured samples were higher than limit for Pb established by Health Canada regulation	Söğüt et al., 2016 [21]
Eighty-eight colors of 25 brands (49 products) of **eye shadows** were	As, Cr, Co, Ni and **Pb**	**GFAAS**	2000	Finland	150 mg of each sample was digested with 5 mL of concentrated nitric acid and 5	**Pb** ranged from < DL to 16.8 ppm	66 out of 88 (75%) of the colours contained more than 5 ppm of at least one element, and all 49	Sainio et al., 2000 [15]

acquired in stores in Helsinki in October 1997. 2 eye shadows were randomly selected for analysis from each cosmetic series.					mL of concentrated hydrochloric acid on a hot plate until the sample was dried. After cooling 2.5 mL of both acids were added and the samples were re-heated until the production of acid fumes ceased. The residues were dissolved in an ultrasonic bath for 30 min into 2 mL of 65% nitric acid and rinsed with water into 10 ml volumetric flasks filling to the mark.		products contained more than 1 ppm of at least one element. In one color the amount of all elements was less than 1 ppm..	
Different samples of **eye shadow** (7 brands, 20 colours) imported from China were randomly selected from products available in the shops at Zaria, Kano and Kaduna markets in Nigeria.	**Pb**, **Cd**, Ni, Cu, Zn, Cr, Co and Mn	**FAAS**	2010	Nigeria	Samples of eye shadow were homogenized in acid washed mortar and pestle. 300 mg of the processed sample was weighed for acid digestion. The sample was brought to a final volume of 50 mL with deionized water.	**Pb** ranged from < DL to 55 µg/g **Cd** ranged from < DL to 8.89 µg/g	2 brands (3 colours) have lead content higher than 20 µg/g (proposed acceptable level). 7 out of 20 colours (35%) of the brands of eye shadows contain cadmium at low concentrations. Use of facial cosmetics like eye shadow exposes users to low levels of heavy metals.	Omolaoye et al., 2010 [9]
Forty samples of **eye shadow**. Samples were divided into five colour categories: pinks, blues, greens, browns, and grays. For each color category, eight popular local and	Cr, **Pb**	**GFAAS**	2017	Malaysia	0.2 g of the eye shadow samples was weighted, then it was placed into a 100 mL Pyrex glass beaker where 4 mL concentrated HNO_3 was added. Left at room temperature for 4 h before placing in the oven overnight at 85°C. After digestion, the sample was taken out and	**Pb** ranged from 14.2 to 284.4 µg/kg	Lead concentrations in all the samples were within the permitted levels stated by the international standards in cosmetics intended for external use. The highest Pb concentration in the	Lim et al., 2017 [22]

imported brands of eye shadows used by consumers were purchased at the local drugstores (Watsons and Guardian) and markets (low-cost shops, cosmetic shops, and street vendors).					allowed to cool at room temperature. Next 1 mL of 30% H_2O_2 was added and the sample was heated for another hour at 85 °C. Then, the clear supernatant was filtered into a volumetric flask and brought to a final volume of 10 mL.		blue color category was found.	
Fifty samples of lipstick (35 samples: 5 colors in 7 brands) and **eye shadow** (15 samples: 3 colors in 5 brands) were selected taken from large cosmetic stores in Isfahan (Iran). The colors studied for each brand of eye shadow were blue, green, and golden.	**Pb**, **Cd**	**GFAAS**	2013	Iran	One gram of each sample was placed into a 100 mL Pyrex glass beaker and digested with 5 mL of concentrated nitric acid on a hot plate at 80◦ C until it dried. The digestion process was repeated twice. Then, 1 mL of concentrated H_2O_2 was added. The residual material was diluted with deionized water to the final volume of 50 mL.	**Pb** ranged from 0.85 to 6.90 μg/g<br>**Cd** ranged from 1.54 to 55.59 μg/g	The concentration of lead in the cosmetics under study was lower than that of FDA standards, however, the amount of cadmium in the analyzed cosmetics was much higher than the standard.<br>Golden color of eye shadow have a higher concentration of cadmium. The continuous use of these cosmetics can increase the absorption of heavy metals especially Cd and Pb into the body when swallowing lipsticks or through dermal cosmetic absorption.	Nourmoradi et al., 2013 [11]
120 samples of 15 brands of pressed powder **eye shadows** in main	**Pb**, **Cd**	**FAAS**	2013	Iran	2 gram of each sample was weighed and were analyzed according	**Pb** ranged from 0.5986 to 202.058 mg/kg	Result reveals that the type of pigment used in eye shadows contributes to its heavy	Mousavi et al., 2013 [7]

seven colors: pink, white, violet, brown, golden, green and blue made from different countries were randomly purchased in local market in Teheran in 2012. Most of the products were imported into Iran (China, Turkey, England, Korea)					to standardized international protocols by wet digestion method, Using HNO_3 and $HClO_4$ and H_2O_2 (3:1:1).	**Cd** ranged from 0.52 to 1066 mg/kg	metal content. Lead and cadmium detected in all colors in wide range of concentrations. The brown and golden colors in all brands have the highest concentrations of lead, while blue and green colors have the lowest lead content. Golden and blue color have the highest and lowest concentration of cadmium respectively.	
Twenty-one popular international **eye shadow** brands from China, France, Italy and Ireland were purchased in Riyadh markets of Saudi Arabia. Eye shadows were categorized according to their price range: low, medium and high.	**Pb**, **Cd**, As and Hg	**GFAAS** (Pb, Cd, As), **FAAS** (Hg)	2017	Saudi Arabia	0.25 g of eye shadow sample was weighed and 8.0 mL of 69% HNO_3, 1.0 mL of 35% H_2O_2, 1.0 mL of 48% HF and 1.0 mL of 36% HCl were added to the sample. Digested eye shadow samples were filtered. After cooling, the extracted samples were transferred to a 50 mL volumetric flask and adjusted with deionized water	**Cd** ranged from 0.42 to 3.92 μg/kg **Pb** ranged from 18.55 to 181.90 μg/kg	Concentration of Cd was fairly high in cheap eye shadow samples. Pb concentration was higher in expensive eye shadows samples.	Ahmed et al., 2017 [23]
Twenty powdered **eye shadows** of different colors were divided into five sample groups, which represented five different brands. Eye	**Pb**, **Cd**, Co, Cr and Ni	**FAAS** (Pb), **ICP-MS** (Cd, Cr, Ni, Co)	2012	Italy	To 1 g of sample, 5 mL of 67% HNO_3 and 1 mL of 40% HF were added into PTFE-vessel. Sample was irradiated with microwaves. After cooling, the sample was transferred in a 50-mL volumetric flask and	**Pb** ranged from 0.25 to 81.5 μg/g **Cd** ranged from 0.6 to 33.04 ng/g	The levels of cadmium, was always low and within acceptable and safe concentrations In case of lead, samples manufactured in China had the safety standards exceeded.	Volpe et al., 2012 [14]

shadows were purchased in store in Benevento (Campania, Italy) but were manufactured in different countries (China, Italy, and USA)					adjusted with deionized water.			
150 cosmetic products of 12 types (**eye shadows**, blushes, lipsticks, three types of lotions, mascaras, foundations, body powders, compact powders, shaving creams, and face paints) were purchased by Frontier from April 22, 2011 to August 16, 2011 on the U.S. market. Including 30 eye shadows from 19 manufacturers.	As, **Cd**, Cr, Co, **Pb**, Hg and Ni	**CVAFS** (Hg), **ICP-MS** (As, Cd, Cr, Co, Pb, Ni)	2014	United States of America	0.5 g of samples was weighed. 25 ml of a 3:1 HNO_3:HF mixture and 3 ml of HCl was added and oven 12h at 130°C. After cooling, the sample was transferred in a 50-mL volumetric flask and adjusted with deionized water.<br><br>For mercury analysis, 4 ml of the analytical solution to another centrifuge tube was transfer and diluted to 40 ml with 5% (v/v) bromine monochloride and heated on a hot plate at no more than 200°C until the volume is reduced to 2–3 ml. Next 25 ml of concentrated HNO_3 added and the evaporation process to a volume of 2–3 ml was repeated. Acid addition and evaporation process was repeated two more times, than the sample was transferred in a 50 mL volumetric flask and	**Pb** ranged from 0.045 to 14 mg/kg (median value 4.0 mg/kg) **Cd** ranged from < DL to 0.36 mg/kg (median value < DL)	Significantly higher lead in eye shadows, blushes, and compact powders, when compared with other cosmetic types, were found in products with high solid filler (such as clay or talc) and pigment content, suggesting that the contaminants may originate in the mineral components.	Hepp et al., 2014 [24]

					adjusted with deionized water.			
Twenty-six different cosmetic products collected from Jordanian market (**eye shadow** – 19 samples from 5 brand; lipstick – 3 brands; lip-gloss – 2 brands; mascara – 3 brands; 3 kohl brands; foundations – 4 brands; hair colour – 2 brands; henns – 2 brands; eyeliner – 3 brands)	**Ag**, As, **Ba**, Be, **Bi**, Ca, **Cd**, Co, Cr, Cu, Mn, Mo, Ni, **Pb**, Sn, Sb, **Sr**, Ti, U, **Tl**, Zn, Zr, Nb, Rb, Sn, Al, Bi, Ca, Fe, K, Mg, Na, P, Si, Th, V and Se	**ICP-OES** (Al, Ba, Bi, Ca, Cd, Co, Cr, Cu, Fe, K, Mg, Mn, Na, Ni, P, Pb, Si, Sr, Th, Ti, U, and V), **ICP-MS** (Ag, As, Ba, Be, Cd, Co, Cr, Cu, Mn, Mo, Ni, Pb, Sn, Sr, Ti, Tl, U, Zn, Zr, Nb, Rb, Sb, and Se)	2015	Jordan	0.25 g of samples was weighed. 2 mL concentration of hydrofluoric acid (HF), 8 mL concentration of HNO_3 and 2 mL H_2O_2 (30%) were added. After digestion process using ultrawave system, samples were transferred into 50 mL flask. Next the samples were treated with 4% boric acid to eliminate the excess of HF and adjusted with deionized water.	**Results from ICP-MS were as follows:** **Ag** ranged from < 1.704 ppm to 2.217 ppm **Ba** ranged from 354.841 ppm to 1183.143 ppm **Bi** was not determined in eye shadows by ICP-MS technique **Cd** ranged from < 0.087 ppm to < 0.121 ppm **Pb** ranged from 6.072 ppm to 12.874 ppm **Sr** ranged from 5.168 ppm to 110.628 ppm **Tl** ranged from 4.133 ppm to 7.956 ppm **Results from ICP-OES were as follows:** **Ag** was not determined in eye shadows by ICP-OES technique **Ba** ranged from 140.93 ppm to 4724.62 ppm **Bi** ranged from 19.87 ppm to 97.36 ppm **Cd** ranged from 1.35 ppm to 7.99 ppm	All brands of eye shadow in the current study had high Pb concentration. Some samples of eye shadows Cd with high concentrations.	Farrag et al., 2015 [1]

						**Pb** ranged from 18.45 ppm to 153.89 ppm<br>**Sr** ranged from 13.75 ppm to 37.75 ppm<br>**Tl** was not determined in eye shadows by ICP-MS technique		
Different brands of anti-freckle creams (3), **eye shadows (3)**, eyeliners (3), facial powders (3), foundation (3), lipsticks (3) and henna (7) were purchased from local markets in Egypt, and Saudi Arabia. All the samples were purchased by different commercial suppliers located in the local markets of both countries	**Pb**, As, **Cd**, **Ag**, **Ba**, Al, Cr, Mn, Co, Ni, Cu and Zn	**ICP-MS**	2016	Egypt	0.1 – 0.2g of each cosmetic sample was weighed. 7.0 mL of the acid mixture Nitric/ Hydrofluoric/hydrochloric acids (HNO_3 /HF/HCl, 4.5:2:0.5) was introduced. After digestion process using ultrawave system, the mixture heated on a hotplate (120 °C) for 30 minutes to drive off the residual HF and HCl transferred into volumetric flask and diluted up to 50 mL	**Pb** ranged from 6.750 to 12.030 mg/kg<br>**Cd** ranged from 0.692 to 1.58 mg/kg<br>**Ag** ranged from 0.157 to 2.183 mg/kg<br>**Ba** ranged from 21.30 to 66.15 mg/kg	The mean concentration of some studied metals as lead, was higher than permissible levels for cosmetics in some of the studied samples. Cadmium and silver were detected.	Issa et al., 2016 [13]
Seventy samples (39 cheap blushers and 31 cheap **eye shadow** samples) belonging to different brands and sources were	**Pb**, Mn, **Cd**, **Ag**, Au, Cu, Cr, Ni, **Ba**, Fe, Al, Zn, and Ti	**ICP-MS**	2019	Saudi Arabia	Samples were digested with concentrated nitric acid before the measurement.	**Ag** ranged from < DL to 137.62 mg/kg<br>**Ba** ranged from 52.18 mg/kg to 267 mg/kg<br>**Cd** ranged from < DL to 3.22 mg/kg<br>**Pb** ranged from < DL to 49.23 mg/kg	Pb and Cd were absent in most samples in turn Ag was detected only in one sample (blue). However women must avoid using cheaper brands to prevent exposure to the high	Zainy et al., 2019 [25]

purchased from local markets of Jeddah, Saudi Arabia, and Cairo, Egypt. The sources of these products differ such as KSA (6), USA (7), Turkey (5), China (13) and Egypt (0). 10 cheap brands of eye shadows were used with six colors (blue – 6; gold – 6; green – 6; pink – 3; violet – 4; white – 6). All samples were available at cheap prices ranging from 0.66 $ up to 20 $.							concentrations of toxic materials which cause a negative effect on human health.	
23 products of assorted brands of eyeshadows, obtained from the local market	K, S, Cl, Al, Si, Ca, Ti, Mn, Fe, **Pb**, Zn, Rb, and **Bi**	**EDXRF**	2018	Brazil	No special sample preparation was needed.	**Pb** ranged from 8 µg/g to 75 µg/g (detected only in two out of 23 samples) **Bi** ranged from 8 µg/g to 139 µg/g (detected only in two out of 23 samples)	Some samples which were claimed as having same composition and produced by the same manufacturer, did not present the same composition based on Cluster Analysis. Relations among 6 eyeshadows' colors and chemical compositions were discovered by using decision trees, where the most determinant elements were Mn, S, Cl, Ca, and Fe. The strongest	Santos et al., 2018 [26]

							correlation was found between the color intensity and the concentration of Mn.	

Abbreviations:

Flame Atomic Absorption Spectrometry (FAAS)

The High-Resolution Continuous Source Graphite Furnace Atomic Absorption Spectrometry (HR-CS GF AAS)

Line-source Graphite Furnace Atomic Absorption Spectrometry (LS GF AAS)

Inductively Coupled Plasma with Mass Spectrometry (ICP-MS)

Inductively Coupled Plasma Optical Emission Spectrometry (ICP-OES)

Potentiometric Stripping Analysis (PSA)

X-Ray Fluorescence Spectrometry (XRFS)

Energy Dispersive X-ray Fluorescence (EDXRF)

Cold Vapor Atomic Fluorescence Spectrometry (CVAFS)

Main ingredients of eye shadows

Table S2. Characteristics of the main ingredients of eye shadow [S1-S8].

Component	Content [%]	Function	Properties
**talc**	60 - 80	• adsorbent<br>• anti-caking agent<br>• enables easier distribution of the product<br>• gives a specific transparency	• greasy to the touch<br>• can be scratched with a fingernail<br>• after grinding, it forms a slippery powder with a slight gloss<br>• good absorbent properties<br>• chemically inert
**mica**	30	• gives silkiness and grip	• transparency
**titanium oxide**	> 25	• filler<br>• UV filter<br>• enables easier distribution of the product<br>• pigment<br>• gives whiteness	• odorless substance
**zinc oxide**	14	• UV filter increases coverage and adhesion<br>• pigment<br>• filler	• disinfectant and bacteriostatic substance<br>• in the non-micronized form, it is a white, amorphous powder<br>• in a micronized form it is transparent<br>• amphoteric properties
**micronized silk powder**	11	• improves the application of shadows on the eyelid<br>• gives silkiness	• hygroscopic moisturizing substance that allows it to absorb water from the environment
**calcium or magnesium carbonate**	> 5	• better coverage<br>• absorbs moisture and sebum<br>• gives mattness<br>• carrier of aromatic substances ($MgCO_3$)	• hygroscopic
**magnesium or zinc stearate**	2 - 10	• increases the grip and spreading of shadows<br>• extends durability<br>• makes the application easier<br>• changes the empty weight	• does not dissolve in water<br>• delicate crystalline form<br>• hydrophobic
**rice powder**	9	• filler<br>• gives silkiness	• electrostatics

		• enables easier distribution of the product	
**kaolin**	5 - 10	• absorbs moisture and fat<br>• filler<br>• enables easier distribution of the product<br>• improves the adhesion of the product<br>• reduces excessive shine	• consists of 45% silicon dioxide<br>• adsorbent<br>• insoluble in water<br>• swells and becomes more plastic in a small amount of water, and becomes more plastic with more water<br>• absorbent<br>• stains well
**pigment**	2 - 30	• opaque coloring matter	• well fragmented<br>• insoluble

[S1] Arct, J., Pytkowska, K., Barska, K., Kifert, K., Pauwels, A. *Leksykon surowców kosmetycznych (Eng. Lexicon of cosmetic raw materials)*, Wydawnictwo Wyższej Szkoły Zawodowej Kosmetyki i Pielęgnacji Zdrowia w Warszawie, Warszawa **2010.**

[S2] Barel, A.O., Paye, M., Maibach, H.I. *Handbook of Cosmetic Science and Technology*, CRC Press, **2014**.

[S3] Glinka, R., Glinka M. *Receptura kosmetyczna z elementami kosmetologii (Eng. Cosmetic recipe with elements of cosmetology)*, MA, Łódź **2008**.

[S4] Welcomme, E., Walter, P., Van Elslande, E., Tsoucaris, G., Investigation of white pigments used as make-up during the Greco-Roman period. *Appl. Phys.* **2006**, A 83, 551–556.

[S5] Salvador, A., Chisvert, A. *Analysis of cosmetic products*. Elsevier Science, **2007**.

[S6] Molski, M. *Chemia piękna (Eng. Beaty chemistry)*, PWN, Warszawa **2009**, 59, 61, 77-78.

[S7] Marzec, A. *Chemia kosmetyków (Eng. Cosmetics chemistry)*, Dom Organizatora, Toruń **2011**, 230-23.

[S8] Jurkowska, S. *Surowce kosmetyczne (Eng. Cosmetics raw materials)*, Ośrodek Informatyczno-Badawczy Ekoprzem, Dąbrowa Górnicza, **2001**, 51-58.

The presence of studied elements in eye shadows vs. the regulation on cosmetics products

Table S3. Source and application of metallic impurities in eye shadows products [S9-S16, 30].

Element	Source and application	Remarks
**Ag**	Silver especially in the form of nanoparticles is widely used in cosmetics due to it significant antiseptic and antimicrobial properties. Silver nanoparticles can also protect some skin disease like atopic dermatitis. Due to antibacterial properties of silver nanoparticles it can be used as preservatives in cosmetics, and in anti-acne preparation. For example, silver nanoparticles, which have antibacterial activity, are also being incorporated into toothpastes and shampoos as preservatives.	Silver chloride deposited on titanium dioxide is on the list of preservatives allowed in cosmetics products. However, 20% AgCl (w/w) on TiO_2 should not be used in products for children under 3 years of age, in oral products and in eye and lip products.<br><br>Silver is on the list of colorants allowed in cosmetic products. Silver nitrate (only for colouring eyelashes and eyebrowns) is on the list of substances which cosmetic products must not contain except subject to the restrictions.
**Ba**	Barium sulfate is used as an opacifying agent in cosmetic products. Cosmetic products containing barium sulfate may be applied to the skin and nails or, incidentally, may come in contact with the eyes and mucous membranes. Products containing these ingredients may be applied as frequently as several times per day and may come in contact with the skin or hair for variable periods following application. This substance is used at concentrations up to 15% in cosmetic products that are sprayed (perfumes) and at concentrations up to 15.8% in powders (face and dusting powders). Because this ingredient is used in products that are sprayed and in powders, it could be incidentally inhaled.	Barium salts, with the exception of barium sulphide and of barium sulfate, lakes, salts and pigments prepared from colouring agents are on the list of substances prohibited in cosmetic products.<br><br>On the list of colorants allowed in cosmetic products are: 1-[(2-Chloro-4-nitrophenyl)azo]-2- naphthol and its insoluble barium, strontium and zirconium lakes, salts and pigments (red); sodium 4-[(2-hydroxy-1-naphthyl)azo]benzenesulphonate and its insoluble barium, strontium and zirconium lakes, salts and pigments (not to be used in eye products) (orange); barium bis[4-[(2-hydroxy-1- naphthyl)azo]-2-methylbenzenesulphonate] (red); sodium 2-[(2- hydroxynaphthyl)azo]naphthalenesulphonate and its insoluble barium, strontium and zirconium lakes, salts and pigments (red); disodium 3-hydroxy-4-[(4-methyl-2- sulphonatophenyl)azo]-2-naphthoate and its insoluble barium, strontium and zirconium lakes, salts and pigments (red); disodium 4-[(5-chloro-4-methyl-2- sulphonatophenyl)azo]-3-hydroxy-2- naphthoate and its insoluble barium, strontium and zirconium lakes, salts and pigments (red); disodium 6-hydroxy-5-[(4- sulphonatophenyl)azo]naphthalene-2- sulphonate and

		its insoluble barium, strontium and zirconium lakes, salts and pigments (yellow); trisodium 1-(1-naphthylazo)-2- hydroxynaphthalene-4′,6,8-trisulphonate and its insoluble barium, strontium and zirconium lakes, salts and pigments (red); disodium 5-amino-4-hydroxy-3- (phenylazo)naphthalene-2,7-disulphonate and its insoluble barium, strontium and zirconium lakes, salts and pigments (red); trisodium 5-hydroxy-1-(4-sulphophenyl)- 4-((4-sulphophenyl)azo)pyrazole-3-carboxylate and its insoluble barium, strontium and zirconium lakes, salts and pigments (yellow); ethanaminium, N-(4-((4- (diethylamino)phenyl)(5-hydroxy-2,4- disulfophenyl)methylene)-2,5- cyclohexadien-1-ylidene)-N-ethyl-, hydroxide, inner salt, calcium salt (2:1) and its insoluble barium, strontium and zirconium lakes, salts and pigments (blue); 4′,5′-dibromo-3′,6′-dihydroxyspiro[isobenzofuran-1(3H),9′- [9H]xanthene]-3-one and its insoluble barium, strontium and zirconium lakes, salts and pigments (orange); disodium 2-(2,4,5,7-tetrabromo-6-oxido3-oxoxanthen-9-yl)benzoate and its insoluble barium, strontium and zirconium lakes, salts and pigments (red); 3,4,5,6-tetrachloro-2-(1,4,5,8- tetrabromo-6-hydroxy-3-oxoxanthen-9- yl)benzoic acid and its insoluble barium, strontium and zirconium lakes, salts and pigments (red); disodium 2-(2,4,5,7-tetraiodo-6-oxido-3- oxoxanthen-9-yl)benzoate and its insoluble barium, strontium and zirconium lakes, salts and pigments (red); barium sulfate (white).
**Bi**	Bismuth oxychloride found wide application in cosmetics as a pearlescent pigment. This substance gives the skin a white, pearly color. Additionally, it shows very good covering properties. Due to its antibacterial properties, it protects the skin against infections. It also facilitates the application of the cosmetic on the skin.	Bismuth chloride oxide (white) is on the list of colorants allowed in cosmetic products.

**Cd**	Cadmium is a deep yellow to orange pigment and mostly present in lipsticks and face powders. The use of cadmium in cosmetics products are due to its color property as it has been used as a color pigment in many industries.	Cadmium and its compounds are on the list of substances prohibited in cosmetic products.
**Pb**	Lead occurs naturally in the environment. Iron oxides are colored pigments used in a variety of color cosmetics that, are mined from the ground, and therefore contain traces of lead.	Lead and its compounds are on the list of substances prohibited in cosmetic products.
**Sr**	Strontium is used in cosmetics in the form of compounds such as: strontium chloride hexahydrate, strontium acetate hemihydrate, strontium hydroxide as well as strontium peroxide. However, the concentrations of these compounds are limited. They mainly serve as buffering agents as well as provides cosmetic effects to the oral cavity (cleaning, deodorization and protection), helps lighten the discomfort of the skin or scalp.	Barium salts, with the exception of barium sulphide and of barium sulfate, lakes, salts and pigments prepared from colouring agents are on the list of substances prohibited in cosmetic products.<br><br>On the list of colorants allowed in cosmetic products are:<br><br>1-[(2-Chloro-4-nitrophenyl)azo]-2- naphthol and its insoluble barium, strontium and zirconium lakes, salts and pigments (red); sodium 4-[(2-hydroxy-1-naphthyl)azo]benzenesulphonate and its insoluble barium, strontium and zirconium lakes, salts and pigments (not to be used in eye products) (orange); barium bis[4-[(2-hydroxy-1- naphthyl)azo]-2-methylbenzenesulphonate] (red); sodium 2-[(2- hydroxynaphthyl)azo]naphthalenesulphonate and its insoluble barium, strontium and zirconium lakes, salts and pigments (red); disodium 3-hydroxy-4-[(4-methyl-2- sulphonatophenyl)azo]-2-naphthoate and its insoluble barium, strontium and zirconium lakes, salts and pigments (red); disodium 4-[(5-chloro-4-methyl-2- sulphonatophenyl)azo]-3-hydroxy-2- naphthoate and its insoluble barium, strontium and zirconium lakes, salts and pigments (red); disodium 6-hydroxy-5-[(4- sulphonatophenyl)azo]naphthalene-2- sulphonate and its insoluble barium, strontium and zirconium lakes, salts and pigments (yellow); trisodium 1-(1-naphthylazo)-2- hydroxynaphthalene-4',6,8-trisulphonate and its insoluble barium, strontium and zirconium lakes, salts and pigments (red); disodium 5-amino-4-hydroxy-3- (phenylazo)naphthalene-2,7-disulphonate and

		its insoluble barium, strontium and zirconium lakes, salts and pigments (red); trisodium 5-hydroxy-1-(4-sulphophenyl)- 4-((4-sulphophenyl)azo)pyrazole-3-carboxylate and its insoluble barium, strontium and zirconium lakes, salts and pigments (yellow); ethanaminium, N-(4-((4- (diethylamino)phenyl)(5-hydroxy-2,4- disulfophenyl)methylene)-2,5- cyclohexadien-1-ylidene)-N-ethyl-, hydroxide, inner salt, calcium salt (2:1) and its insoluble barium, strontium and zirconium lakes, salts and pigments (blue); 4′,5′-dibromo-3′,6′-dihydroxyspiro[isobenzofuran-1(3H),9′- [9H]xanthene]-3-one and its insoluble barium, strontium and zirconium lakes, salts and pigments (orange); disodium 2-(2,4,5,7-tetrabromo-6-oxido3-oxoxanthen-9-yl)benzoate and its insoluble barium, strontium and zirconium lakes, salts and pigments (red); 3,4,5,6-tetrachloro-2-(1,4,5,8- tetrabromo-6-hydroxy-3-oxoxanthen-9- yl)benzoic acid and its insoluble barium, strontium and zirconium lakes, salts and pigments (red); disodium 2-(2,4,5,7-tetraiodo-6-oxido-3- oxoxanthen-9-yl)benzoate and its insoluble barium, strontium and zirconium lakes, salts and pigments (red).
**Tl**	Cosmetic products may contain either petroleum or mineral-based colorants. Both petroleum and mineral-based pigments may contain heavy metals, which also includes thallium. Additionally, thallium acetate as a compound causes hair loss, was the active principle in "koremlu," a widely advertised depilatory which brought many of its victims almost to the point of death. The concern making this product was forced into bankruptcy in 1932 by numerous damage suits.	Thallium and its compounds are on the list of substances prohibited in cosmetic products.

[S9] Kantorová, V., Loula, M., Kaňa, A., Mestek, O., Determination of silver nanoparticles in cosmetics using single particle ICP-MS, *Chemical Papers*, **2021**, 75, pp. 5895–5905.
[S10] Shoseyov, O., Levy, I., BioInspired Devices and Materials of the Future, *Nano BioTechnology,* **2008**, 322.

[S11] Kim, K.-J. Antifungal Effect of Silver Nanoparticles on Dermatophytes, *Journal of Microbiology and Biotechnology*, **2008**, 18, pp. 1482-1484.
[S12] Noorbakhsh, F., Antifungal Effects of Silver Nanoparticle Alone and with Combination of Antifungal Drug on Dermatophyte Pathogen Trichophyton Rubrum, *International Conference on Bioscience, Biochemistry and Bioinformatics,* **2011**, 5, pp. 364-367.
[S13] Johnson, W., Bergfeld, W.F., Belsito, D.V., Hill, R.A., Klaassen, C.D. Liebler, D.C., Marks, J. G., Shank, R.C., Slaga, T. J., Snyder, P.W. Gill, L. J., Heldreth, B., Safety Assessment of Barium Sulfate as Used in Cosmetics, *International Journal of Toxicology*, **2018**, 37, pp. 5-11.
[S14] Saleh, F., Saif, R., Murshed, D., Abdulmageed, B. M. A., Determination of Cadmium in Some Cosmetic Products, *Food and Analitycal Methods*, **2020.**
[S15] Borowska, S., Brzóska, M.M., Metals in cosmetics: implications for human health, *Journal of Applied Toxicology*, **2015**, 35, 6, pp.551-572.
[S16] Nelson, F.P., Rumsfield, J., Cosmetics Content and Function, *International Journal of Dermatology*, **1988**, 27(10), pp. 665-672.

Analysis of the certificate reference material of human hair and laboratory reference material of eye shadows sample

Table S4. Results gathered for the certificate reference material of human hair spiked with Tl and Bi standards in such an amount that the final concentration in the studied sample was 60 μg/kg (the data for Bi and Tl are not given in the certificate of the NCS ZC81002b Human Hair) and for sample of eye shadows spiked with Tl and Bi and treated as laboratory reference material (checked independently by the reference technique). Results presented in the table S4 were generated by the ICP-MS technique.

Element	Certificate value ± uncertainty [mg/kg] for NCS ZC81002b Human Hair	Obtained results for the NCS ZC81002b Human Hair sample for three independent measurements ± SD [mg/kg]	Recovery [%]	Obtained results for one chosen eye shadows sample for three independent measurements ± SD [mg/kg]
Ag	0.037 ± 0.002	0.036 ± 0.003	97.3	0.093 ± 0.002
Ba	11.1 ± 1.3	10.68 ± 0.22	96.3	269.3 ± 0.6
Bi (spiked)* 0.06 μg/kg	not given	62.0 ± 4.2	103	61.3 ± 1.2
Cd	0.072 ± 0.01	0.069 ± 0.007	95.8	0.39 ± 0.04
Pb	3.83 ± 0.18	3.74 ± 0.01	97.6	6.35 ± 0.08
Sr	8.17 ± 0.69	7.75 ± 0.04	94.8	37.27 ± 0.08
Tl (spiked)* 0.06 μg/kg	not given	55.3 ± 2.2	92.2	56.4 ± 1.9

*elements not indicated in the certificate

CA analysis

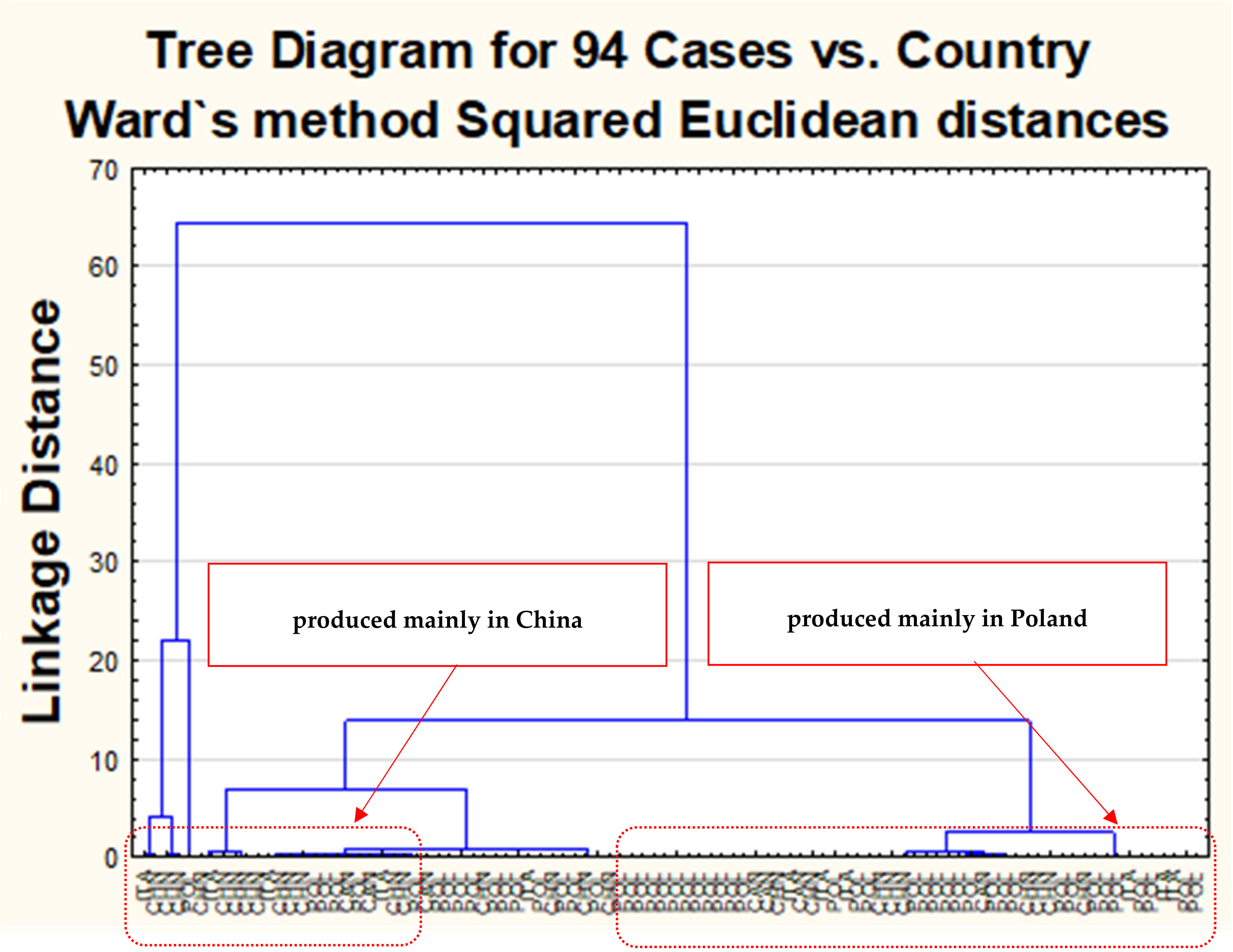

Figure S1. Tree diagram for all studied samples with regard to the country of origin of eye shadows, where ITA – Italy, CHIN – China, POL – Poland, CAN – Canada.

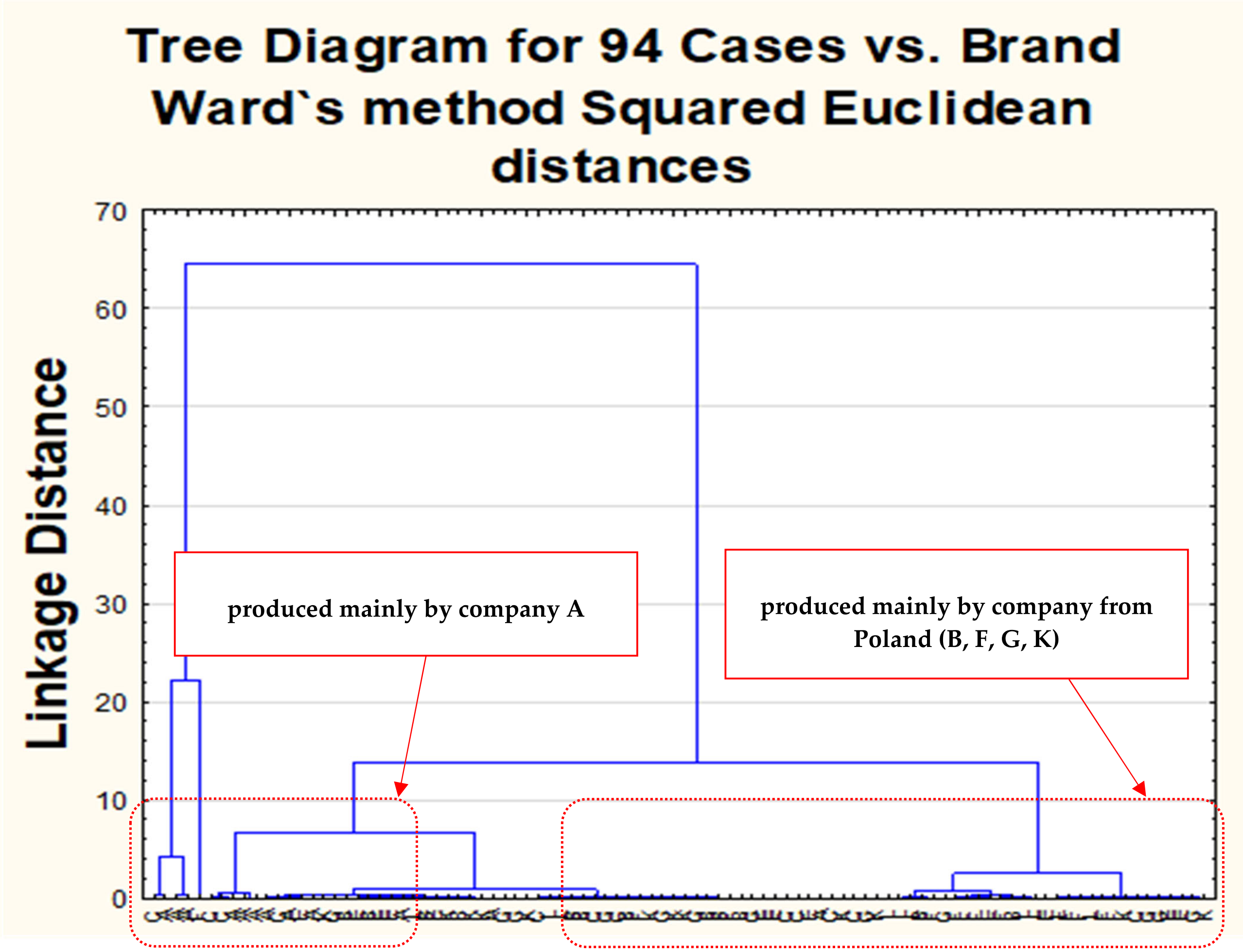

Figure S2. Tree diagram for all studied samples with regard to the producer of eye shadows. In total 8 companies were distinguished: A (China), B (Poland), C (China and Italy); E (China, Italy and Canada), F (Poland), G (Poland), K (Poland), L (China and Poland).

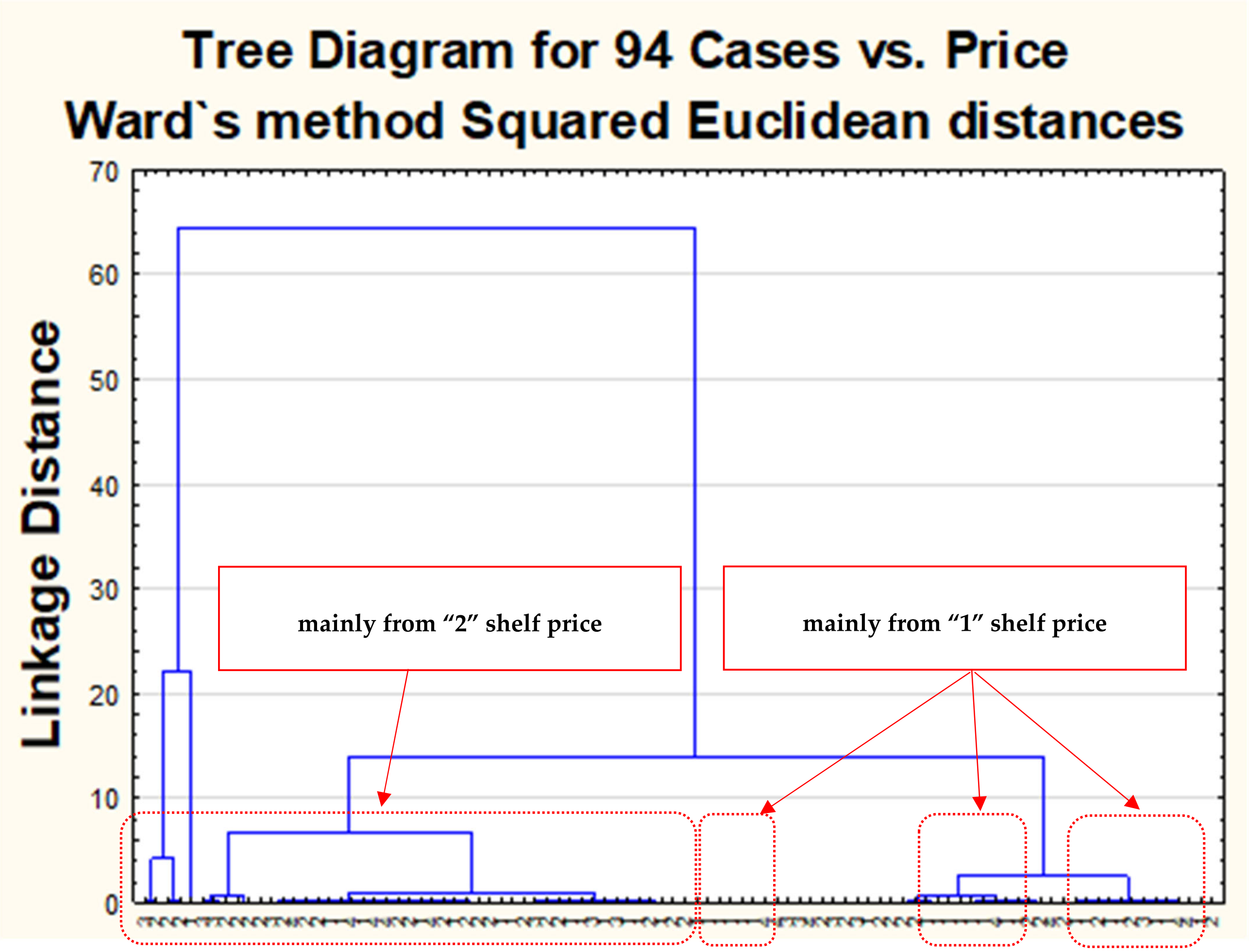

Figure S3. Tree diagram for all studied samples with regard to the price range of eye shadows, where: group “1” - the cheapest products (brands B, F and G), group “2” - medium price products (brands A, K and L), group “3” - expensive (brand C) and group “4” - very expensive (brand E).

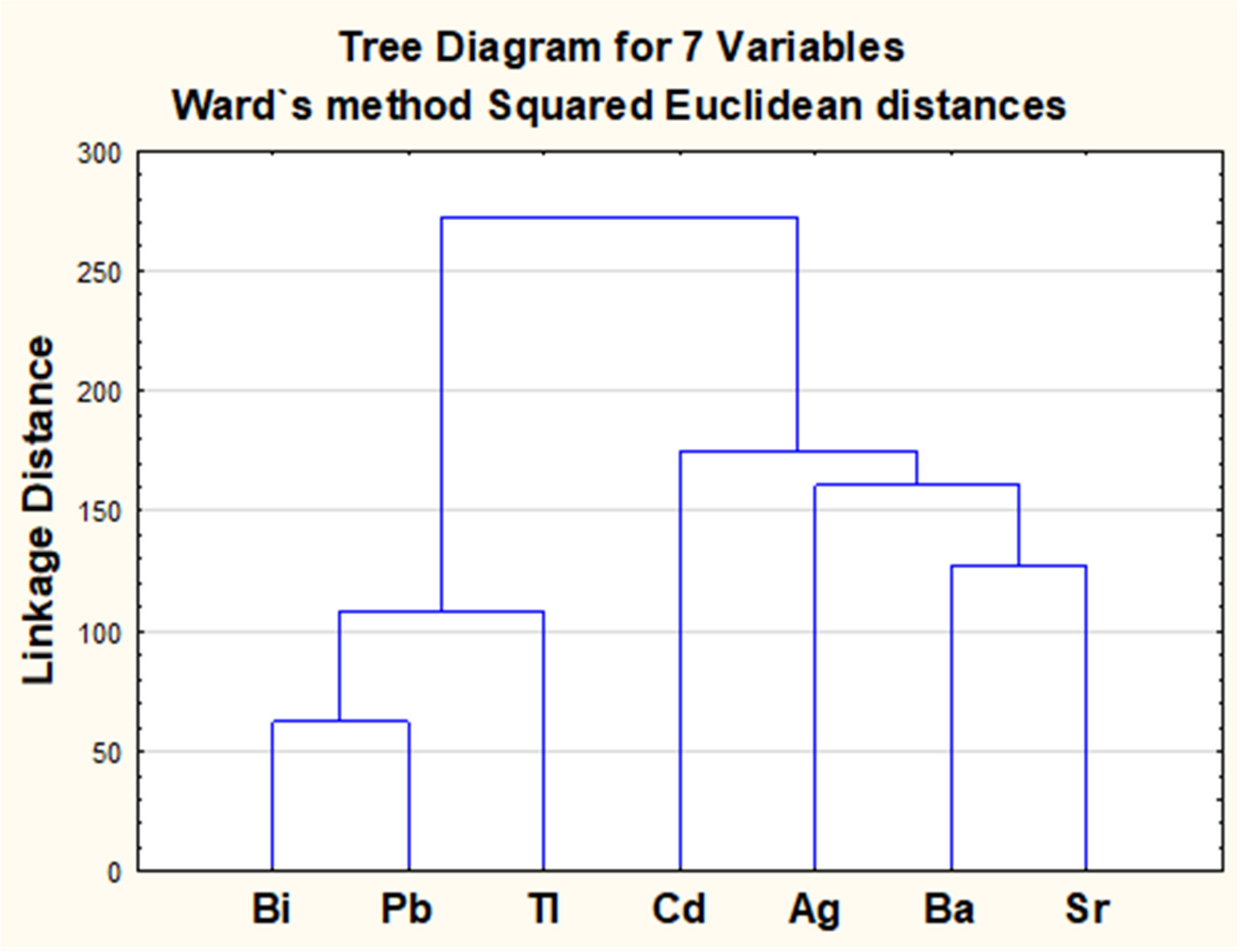

Figure S4. Tree diagram for all studied variables (metallic impurities) in all eye shadows samples.
